# Analysis of global routine immunisation coverage shows disruption and stagnation during the first two-years of the COVID-19 pandemic with tentative recovery in 2022

**DOI:** 10.1016/j.jvacx.2023.100383

**Published:** 2023-09-06

**Authors:** Beth Evans, Olivia Keiser, Laurent Kaiser, Thibaut Jombart

**Affiliations:** a Institute of Global Health, Faculty of Medicine, University of Geneva, Geneva, Switzerland; b Division of Infectious Diseases, Geneva University Hospitals, Geneva, Switzerland; c Geneva Centre for Emerging Viral Diseases, Geneva University Hospitals and Faculty of Medicine, Geneva, Switzerland; d MRC Centre for Global Infectious Disease Analysis, School of Public Health, Imperial College London, UK

**Keywords:** Vaccine, Immunisation, Coverage, Pandemic, COVID-19, Routine immunisation, Modelling, Global health, Time series, Global, Decline, Recovery

## Abstract

Whilst it is now widely recognised that routine immunisation (RI) was disrupted by the COVID-19 pandemic in 2020, and further so in 2021, the extent of continued interruptions in 2022 and/or rebounds to previous trends remains unclear.

We modelled country-specific RI trends using validated estimates of national coverage from the World Health Organisation and United Nation Children’s Fund for 182 countries (accounting for > 97% of children globally), to project expected diphtheria, tetanus, and pertussis-containing vaccine first-dose (DTP1), third-dose (DTP3) and measles-containing vaccine first-dose (MCV1) coverage for 2020–2022 based on pre-pandemic trends (from 2000 to 2019).

We provide further evidence of peak pandemic immunisation disruption in 2021, followed by tentative recovery in 2022. We report a 3.4% (95 %CI: [2.5%; 4.4%]) decline in global DTP3 coverage in 2021 compared to 2000–2019 trends, from an expected 89.8% to reported 86.4%. This coverage gap reduced to a 2.7% (95 %CI: [1.8%; 3.6%]) decline in 2022, with reported coverage rising to 87.2%. Similar results were seen for DTP1 and MCV1. Whilst partial rebounds are encouraging, global coverage decline translates to a 17-year setback in RI to 2005 levels, and the majority of countries retain coverage at or lower than pre-pandemic levels. The Americas, Africa, and Asia were the most impacted regions; and low- and middle-income countries the most affected income groups.

The number of annual Zero Dose (ZD) children – indicating those receiving no immunisations – increased from 12.1 million (M) globally in 2019 to a peak of 16.7 M in 2021, then reduced to 13.1 M in 2022. Overall, we estimate an excess of 8.8 M ZD children cumulatively in 2020–2022 compared to pre-pandemic levels.

This work can be used as an objective baseline to inform future interventions to prioritise and target interventions, and facilitate catch-up of growing populations of under- and un-immunised children.

## Highlights

• Routine immunisation suffered or stagnated in ∼75% of countries during COVID-19.
• Global vaccine coverage partially rebounded in 2022, hinting tentative recovery.
• We quantify a cumulative 8 + million additional Zero Dose children in 2020–2022.

## Introduction

Routine immunisation (RI) is a life-saving global health intervention: each year over 120 million (M) unique children are vaccinated against childhood diseases,^1^ and this prevents an estimated 3.5–5 M deaths per year [1]. It is now widely recognised that RI was disrupted globally during the start of the COVID-19 pandemic in 2020 [2], [3], [4], and continued into 2021 [5], [6], [7]. Single-country studies (e.g., Brazil – [8]), surveys [9], [10], [11], and literature reviews have reported delays and interruptions in service delivery, decreases in attendance, and/or full suspension of services during the pandemic, e.g., during periods of quarantine or implementation other non-pharmaceutical interventions (NPIs). Observational and modelling studies have evaluated and quantified the reduction in immunisation coverage at the country-, regional-, and global-level, with estimates ranging from declines of 2 percentage points (%) [12] to over 7% globally in 2020 [13] and estimates of further declines of 1–3% in 2021 [5], [6], [7]. Disruptions to RI can result in a material increase in susceptible individual populations vulnerable to vaccine-preventable disease (VPD) outbreaks: prior research has established associations between lower vaccination coverage and outbreaks of VPDs *e.g*., Measles outbreaks in Zambia [14] and in the United States of America [15]). Tracking of VPDs globally indicates increasing outbreaks [16], [17], highlighting the potential health implications of the knock-on effects of the pandemic.

Whilst the evidence of RI disruptions during the first two years of the pandemic is now well documented, it remains unclear whether such disruptions continued in 2022 or whether recovery occurred, and how this varied across countries. To our best knowledge, no peer reviewed papers have yet been published on the global perspective of 2022 coverage; however promising signs of recovery in Gavi-eligible countries has been highlighted in the grey literature [18].

Here, we build on previously published methods [19] to test and quantify the extent of RI disruption in 2020 to 2022 compared to pre-pandemic trends using latest datasets (which include retrospective amendments to prior year data). Reaching Zero Dose (ZD) children – those that receive no vaccinations – is seen as essential to achieving the Sustainable Development Goals commitment to “leave no one behind” [20]. Therefore, we also translate modelled coverage disruptions into quantification of under- and un-immunised children, factoring in demographic changes over time. We investigate year-on-year trends within countries, with the aim to facilitate future investigations of the key factors underpinning coverage dynamics for future pandemic preparedness.

## Materials and methods

### Data collection

Building on our previously published methodology to identify deviations from temporal trends in RI [19], we investigated changes in RI coverage using three key indicators: diphtheria, tetanus, and pertussis-containing vaccine first-dose (DTP1) and third-dose (DTP3) coverage, and measles-containing vaccine first-dose (MCV1). DTP1 is typically administered at around six-weeks of age and is used as a proxy for inequity to quantify ZD children [24]. DTP3 is delivered to children aged approximately fourteen-weeks old and serves as a general marker for immunisation system performance, used by national and global immunisation stakeholders. MCV1, typically delivered at nine-months of age, is often used as an additional indicator of health system performance.

COVID-19 was declared as a global pandemic by the World Health Organisation (WHO) on 11th March 2022 and the end of the pandemic emergency on 5th May 2023. We therefore refer to 2020–2022 as the ‘pandemic period’ and 2019 and before as ‘pre-pandemic’.

We used coverage data published by the WHO and United Nation Children’s Fund (UNICEF) Estimates of National Immunisation Coverage (WUENIC [21], [22]) from 2000 to 2022 inclusive, using the latest (July 2023) WUENIC data release [23], [24]. WUENIC estimates are produced following a computational logic-based method [21], based on assessing quality and inclusion of data provided by countries. Whilst WUENIC data offers a standardised and globally-recognised estimate of coverage per country-antigen, lack of or poor-quality data poses challenges for modelling and interpretation – see the ‘Limitations’ section for interpretation guidance.

Population data was sourced from the United Nations World Population Prospects (UNWPP) 2022 release [25]. We use surviving infant (SI) estimates throughout – this refers to the birth cohort minus children that die before their first birthday, and is the standard target population for vaccines delivered in the first year of life. Demographic changes could contribute to estimates of missed immunisations since these are a factor of population and coverage. However, between 2019 and 2022 the annual global SI estimate fell slightly from 134 M to 130 M [25], therefore demographic effects – whilst included – should be minimal at aggregate levels. Income group classification was taken from the World Bank’s categorisation of countries for 2022 [26]. It is noted that some states^2^ have changed income group classification between 2020 and 2022. We have opted to use 2022 classification for simplicity and to reflect economic changes in classification that occurred during the pandemic.

### Statistical analysis

We used Auto Regressive Integrated Moving Average (ARIMA) modelling [27] to capture temporal trends in coverage for each country from 2000 to 2019 to forecast expected coverage in 2020–2022 using the ‘*forecast’* package in R [27]. ARIMA models are characterised by three order terms – *p*, *d*, and *q* – representing the order of the Auto Regressive (AR) term, the number of differencing steps required to make the time series stationary (Integrated, I term), and the order of the Moving Average (MA) term respectively. These terms are selected by:

• Conducting Kwiatkowski-Phillips-Schmidt-Shin tests, with the null hypothesis that the time series is stationary around a deterministic trend against the alternative of a unit root to validate whether the time series is stationary [28]. Where not stationary, each time series is differenced and then re-validated to test if stationary to determine the integrative order, *d*.
• Stepwise algorithm to traverse the model space to select the model with the smallest Akaike Information Criterion (AIC) [29]. AIC scores evaluate a model fit whilst accounting for model complexity, with lower AIC scores indicating better fit. AIC minimisation is used to identify *p* and *q* terms.

Since WUENIC data is annual, seasonal ARIMA models were not required. The inclusion of 2020 and 2021 in the ARIMA model fitting to forecast coverage in later years was ruled out as many countries showed sharp changes in point estimates of coverage in 2020–2021, and we did not wish to bias model selection to fit these data points. While this results in wider confidence intervals (CIs) for later projections, results were essentially unchanged when also including 2020 and 2021 in the model-fitting stage for countries which did not show sharp changes during the pandemic.

193 states have both coverage and income data reported in WUENIC and UNWPP datasets respectively. This initial set of states was reduced to 190 by removing those with incomplete coverage data from 2000 to 2019 (Montenegro, South Sudan, and Timor Leste). A further eight states were excluded prior to investigating global, regional, income group, and year-on-year differences between expected and observed coverage, based on two exclusion criteria:

• **Lack of recent WUENIC coverage updates**: For five countries (the Central African Republic, Haiti, Guinea, Lesotho, and Somalia), WUENIC report an inability to update coverage estimates for multiple years due to the absence of additional information meeting their criteria, resulting in “flatlining” coverage estimates to prior year levels [23].
• **Major (non-COVID-specific) events reportedly affecting immunisation**: including Myanmar [30], Ukraine [31], and the Democratic People’s Republic of Korea (which reported a 12-month stockout at national and subnational levels in 2022 and 0% coverage for all vaccines [32]), so as not to bias results to overestimate pandemic-associated coverage declines.

Changes in coverage were measured as the difference between the WUENIC-reported (referred throughout as ‘reported’) and ARIMA-forecasted (referred throughout as ‘expected’) coverage for a given year. Values are reported as percentages and these refer to percentage point changes e.g.*,* −2% refers to a 2-percentage point difference or decline in coverage, for example from 87% to 85% coverage. The following analyses were completed for each vaccine dose. For all statistical tests, statistical significance threshold of 0.05 was used (*p*-values for results are stated).

• **Global trends:** We explored absolute changes in reported coverage over time per country to quantify coverage changes from 2020 to 2022 worldwide, compared to previous levels. We then conducted *t-tests* of reported vaccination coverage against the null hypothesis of the absence of change in trends from prior years for each year to identify and quantify the global existence of RI disruption and recovery.
• **Income group and region stratification:** We investigated regional and income group-level heterogeneities using linear models with coverage changes as a response variable and Analysis of Variance (ANOVA) using the corresponding categorisation of countries. The combined impact of income group and region was explored through looking at the linear models of both variables combined and conducting ANOVA.
• **Country-level:** We compared the 95% CIs of ARIMA-forecasts to reported coverage. We classify country pandemic impact through assessment of two dimensions: (i) absolute changes in coverage compared to pre-pandemic and (ii) relative performance compared to expected coverage.
• **Un- and under-immunised children, and ZD:** We calculated the number of missed and under-immunised children per year by multiplying WUENIC coverage by SI population estimates per year from UNWPP. We compared numbers of unvaccinated children to 2019-levels. See discussion for potential impact of demographic changes.

All analyses were conducted using R, version 4.3.1 [33] and can be reproduced using a publicly available *reportfactory* including required data and scripts [34].

## Results

After selecting countries with sufficient historical data, and removing countries based on the exclusion criteria described in the methods, we conducted analyses on 182 countries, comprising 127 M SIs in 2022 (97% of the global SI population, 3% smaller SI population than in 2019).

**Fig. 1:**
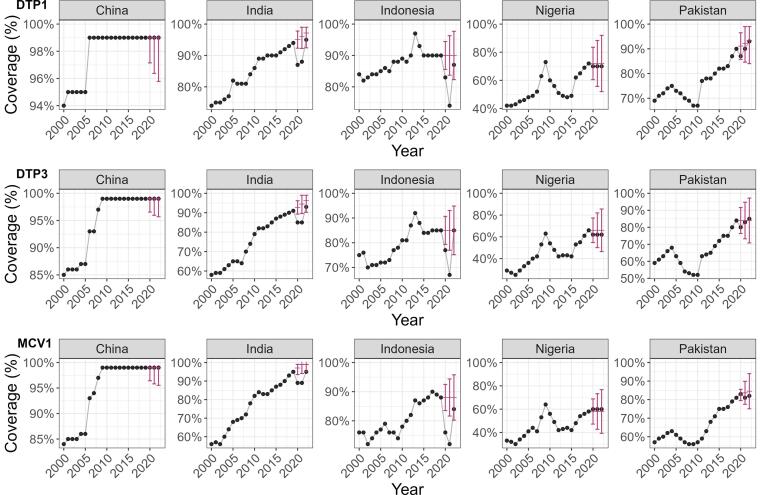
**Example modelling outputs for five largest countries:** Expected (2020–2022) and reported (2000–2022) vaccine coverage for DTP1 (panel A), DTP3 (panel B), and MCV1 (panel C) for 2000–2022. These graphs show WUENIC-reported coverage data (black dots), and the corresponding ARIMA predictions and the associated 95% Confidence Intervals (red bars). DTP1 = diphtheria, tetanus, and pertussis-containing vaccine first-dose, DTP3 = diphtheria, tetanus, and pertussis-containing vaccine third-dose, MCV1 = measles-containing-vaccine first-dose. ARIMA = AutoRegressive Integrated Moving Average modelling. WUENIC = WHO/UNICEF Estimates of National Immunization Coverage. (For interpretation of the references to colour in this figure legend, the reader is referred to the web version of this article.)

Examples of ARIMA models and forecasts are provided for the countries with five largest populations – Fig. 1. Complete results by country and vaccine dose can be seen in the **Appendix S10**.

### Global

**Fig. 2:**
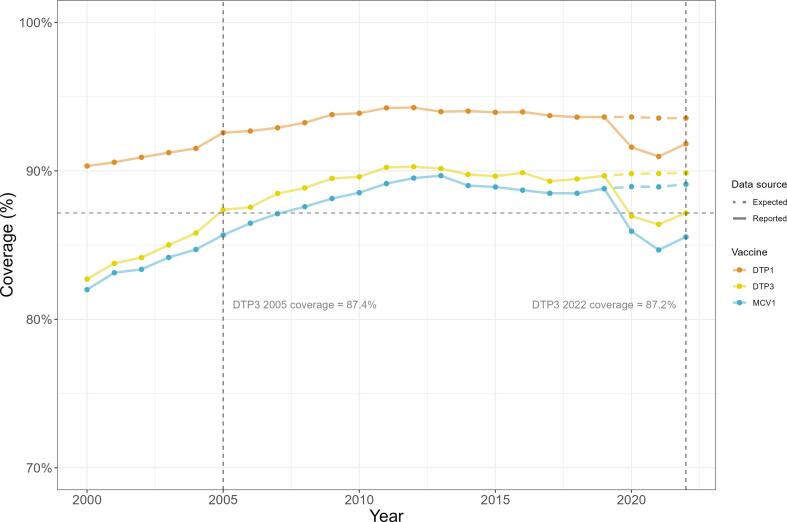
**Global trends in DTP1 (orange), DTP3 (yellow), and MCV1 (blue) coverage over time.** Expected (dotted line) refers to ARIMA-forecast coverage levels. WUENIC-reported data (solid line) is lower than expected coverage during the pandemic from 2020 to 2022. Horizontal and vertical dashed yellow lines compare reported DTP3 2022 coverage to year coverage levels last seen. DTP1 = diphtheria, tetanus, and pertussis-containing vaccine first-dose, DTP3 = diphtheria, tetanus, and pertussis-containing vaccine third-dose, MCV1 = measles-containing-vaccine first-dose. ARIMA = AutoRegressive Integrated Moving Average modelling. WUENIC = WHO/UNICEF Estimates of National Immunization Coverage. (For interpretation of the references to colour in this figure legend, the reader is referred to the web version of this article.)

Global coverage declined year-on-year from 2019 to 2021, and then exhibited a partial rebound in 2022 *i.e.*, 2022 coverage was lower than pre-pandemic but higher than peak pandemic declines. DTP3 coverage fell from 89.7% in 2019, to 87.0% in 2020, and 86.4% in 2021; in 2022 coverage partially rebounded to 87.2%. This trend was seen across all vaccine doses – see Fig. 2. These declines translate to a 17-year regression in immunisation coverage in 2021: DTP3 coverage in 2020 was at a level last observed in 2005, and regressed to close to 2004 coverage levels in 2021, before reverting to 2005 levels in 2022.

Coverage deltas exhibited a parabolic shape with similar deltas in 2020 and 2022 and peak declines in coverage in 2021. The uptick of coverage in 2022 indicates a departure from the continuously declining coverage trends from 2019 to 2021 and hints at the start of a global recovery in RI in 2022– see Table 1.

**Table 1:** Expected and reported global coverage in 2020–2022 for DTP3.

**Year**	**Expected coverage**	**Reported coverage**	**Delta [95% Confidence Intervals]**	***p-*value**	**Coverage last seen in**
2020	89.8%	87.0%	−2.9% [−2.1%; −3.7%]	<0.0001	2005
2021	89.8%	86.4%	−3.4% [−2.5%; −4.4%]	<0.0001	2004
2022	89.9%	87.2%	−2.7% [−1.8%; −3.6%]	<0.0001	2005

Caption: Table details output from conducting a *t-*test on the difference between ARIMA-forecasted (expected) and WUENIC-reported (reported) coverage each year for DTP3. Deltas are the difference between expected and reported coverage, given with 95% confidence intervals from the modelling in square brackets. The final column details when global coverage was last reported at the reported level for that year to indicate years of coverage regression. DTP3 = diphtheria, tetanus, and pertussis-containing vaccine third-dose. ARIMA = AutoRegressive Integrated Moving Average modelling. WUENIC = WHO/UNICEF Estimates of National Immunization Coverage.

Similar trends were seen observed for all vaccines, with MCV1 coverage exhibiting largest declines and least recovery in 2022 – see **Appendix S6** for complete tables.

### Region and income group

There was no evidence of coverage declines versus expectations in all years in Europe and Oceania for DTP. Three regions – Africa, the Americas, and Asia – exhibited lower coverage than expected in 2020 and 2021, with larger coverage reductions in 2021. In 2022 for DTP, both Asia and the Americas reported approximately 2 percentage point improvement in coverage compared to 2020 and 2021– suggesting stronger rebounds in coverage for these regions (Asia exhibited coverage within expected 95% CIs for DTP1 and DTP3, and the Americas for DTP1). In contrast, African coverage remained relatively plateaued at 2021 levels throughout 2022. See Table 2 for ANOVA results per year for DTP3. For MCV1 partial recovery was seen to a lesser extent across the three regions; and there was some evidence of coverage declines for Europe in 2022 (MCV1 Europe delta: −2.5%, 95% CI: −4.7%; −0.3%) – see **Appendix S7**.

**Table 2:** Expected and reported coverage in 2020–2022 for DTP3 by region.

		**2020**				**2021**				**2022**			
**Region**	**Sample size**	**Expected**	**Reported**	**Delta**	**p-value**	**Expected**	**Reported**	**Delta**	**p-value**	**Expected**	**Reported**	**Delta**	**p-value**
**Africa**	49	**84.2%**	**81.5%**	**−2.8%** **[−4.2%; −1.4%]**	**0.00014**	**84.3%**	**80.6%**	**−3.7%** **[−5.4%; −2%]**	**< 0.0001**	**84.4%**	**80.8%**	**−3.6%** **[−5.4%; −1.9%]**	**< 0.0001**
**Americas**	34	**90.5%**	**83.6%**	**−6.9%** **[−8.6%; −5.3%]**	**< 0.0001**	**90.5%**	**83.9%**	**−6.5%** **[−8.6%; −4.5%]**	**< 0.0001**	**90.6%**	**85.4%**	**−5.2%** **[−7.3%; −3.0%]**	**< 0.0001**
**Asia**	45	**92.5%**	**89.2%**	**−3.3%** **[−4.7%; −1.8%]**	**< 0.0001**	**92.6%**	**89.1%**	**−3.5%** **[−5.3%; −1.7%]**	**0.00018**	92.7%	91.1%	−1.6% **[−**3.5%; 0.2%]	0.088
**Europe**	40	94.4%	93.7%	−0.7% [**−**2.3%; 0.8%]	0.37	94.3%	93.2%	−1.2% **[−**3.1%; 0.7%]	0.23	94.3%	93.1%	−1.2% **[−**3.1%; 0.8%]	0.25
**Oceania**	14	85.8%	87.7%	1.9% [**−**0.7%; 4.5%]	0.15	85.6%	84.5%	−1.1% **[−**4.4%; 2.1%]	0.49	85.5%	84.2%	−1.3% **[−**4.6%; 2.1%]	0.45

Caption: Table details output from conducting ANOVA on the difference between ARIMA-forecasted (expected) and WUENIC-reported (reported) coverage each year by region for DTP3. Deltas are the difference between expected and reported coverage, with 95% Confidence Intervals (CIs) in square brackets. Negative deltas mean coverage was lower than expected. Bold rows indicate where reported coverage is outside (lower, in all cases) the expected 95% CIs. DTP3 = diphtheria, tetanus, and pertussis-containing vaccine third-dose. ARIMA = AutoRegressive Integrated Moving Average modelling. WUENIC = WHO/UNICEF Estimates of National Immunization Coverage.

Venezuela's income group is unclassified by the World Bank therefore Venezuela is excluded from income group analyses. All income groups except for high-income countries (HICs) exhibited statistical evidence of declines in coverage for all vaccines during the pandemic for all vaccine doses (except for UMICs for DTP1 in 2022 – see **Appendix S7**). In 2020 the greatest divergence in expected versus reported coverage was seen in upper-middle-income countries (UMICs) for DTP3; whereas in 2021 low (LICs) and lower-middle-income (LMICs) countries exhibited the largest negative coverage deltas. A preliminary rebound was seen in 2022 moreso in LMICs and UMICs than LICs: LIC coverage remained sustained around the lows seen in 2021 – see Table 3 and **Appendix S7**.

**Table 3:** Expected and reported coverage in 2020–2022 for DTP3 by income group.

		**2020**				**2021**				**2022**			
**Income group**	**Sample size**	**Reported**	**Expected**	**Delta**	**p-value**	**Reported**	**Expected**	**Delta**	**p-value**	**Reported**	**Expected**	**Delta**	**p-value**
**LIC**	22	**78.3%**	**81.1%**	**−2.9%** **[−5.1%; −0.7%]**	**0.011**	**76.2%**	**81.1%**	−4.9% [−7.4%; −2.3%]	**0.00021**	**77.1%**	**81.2%**	**−4.1%** **[−6.8%; −1.5%]**	**0.0022**
**LMIC**	48	**83.7%**	**86.9%**	**−3.2%** **[−4.7%; −1.7%]**	**< 0.0001**	**81.9%**	**87.1%**	−5.1% [−6.9%; −3.4%]	**< 0.000**1	**83.4%**	**87.2%**	**−3.8%** **[−5.6%; −2.1%]**	**< 0.0001**
**UMIC**	52	**86.0%**	**90.5%**	**−4.5%** **[−5.9%; −3.0%]**	**< 0.0001**	**86.4%**	**90.3%**	**−3.9%** **[−5.5%; −2.2%]**	**< 0.0001**	**87.3%**	**90.3%**	**−3.0%** **[−4.8%; −1.3%]**	**0.00059**
**HIC**	59	94.2%	95.2%	−1.0% [−2.3%; 0.4%]	0.16	94.3%	95.2%	−0.9% [−2.4%; 0.7%]	0.28	94.5%	95.1%	−0.7% [-2.3%; 0.9%]	0.4

Caption: Table details output from conducting ANOVA on the difference between ARIMA-forecasted (expected) and WUENIC-reported (reported) coverage each year by income group. Deltas are the difference between expected and reported coverage, with 95% Confidence Intervals (CIs) in square brackets. Negative deltas mean coverage was lower than expected. Bold rows indicate where reported coverage is outside (lower, in all cases) the expected 95% CIs. LIC: Low-income Country. LMIC: Lower-middle-income Country. UMIC: Upper-middle-income Country. HIC: High-income Country. DTP3 = diphtheria, tetanus, and pertussis-containing vaccine third-dose. ARIMA = AutoRegressive Integrated Moving Average modelling. WUENIC = WHO/UNICEF Estimates of National Immunization Coverage.

**Fig. 3:**
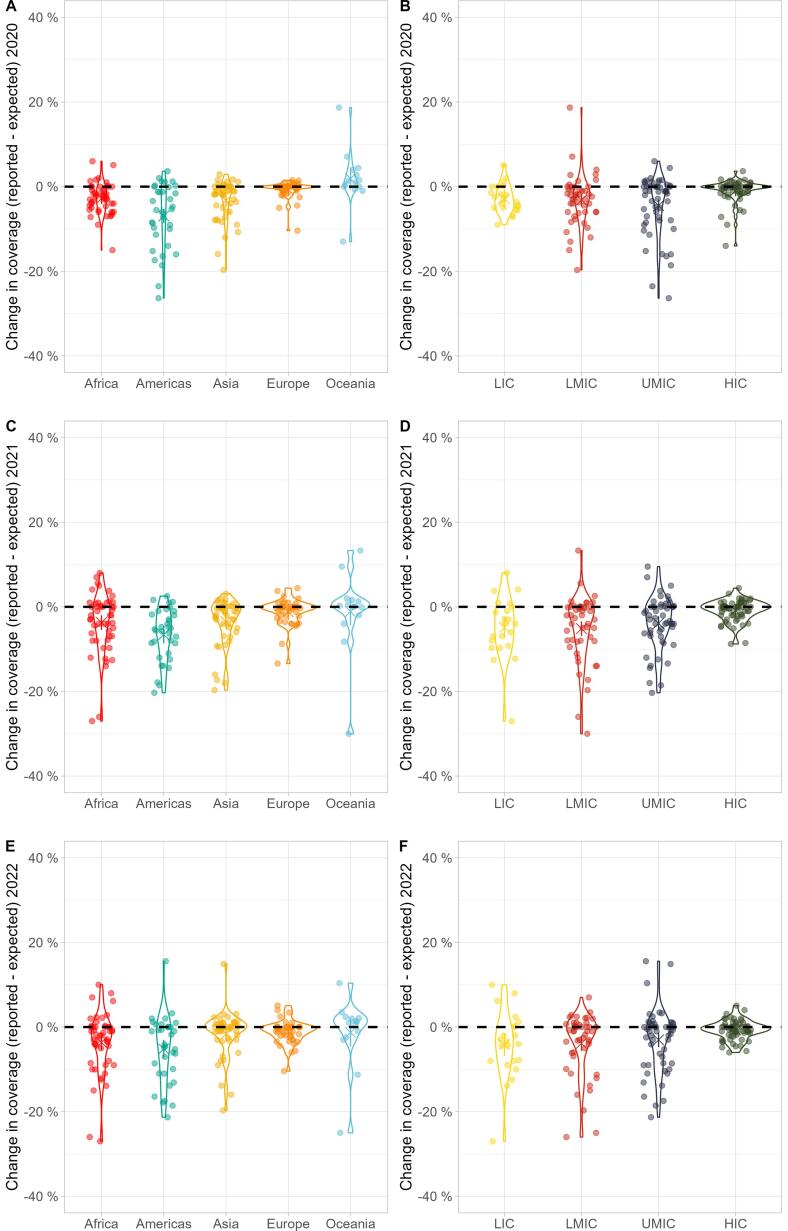
**Differences between expected and reported DTP3 vaccine coverage in 2020**–**2022 by region and income groups.** Points represent individual countries, grouped, and coloured according to (panels A, C & E) region classification and (B, D & F) World Bank income groups, over 2020 (panels A & B), 2021 (panels C & D), and 2022 (panels E & F). Country coordinates on the X-axis were jittered for visibility. Values on the y-axis are indicated as absolute differences between reported and expected vaccine coverage, in percentages. Violin plots show the density of the data within each group: wider lines indicate more datapoints. The black dashed horizontal lines indicate no change in coverage. LIC: Low-income Country. LMIC: Lower-middle-income Country. UMIC: Upper-middle-income Country. HIC: High-income Country. DTP3 = diphtheria, tetanus, and pertussis-containing vaccine third-dose.

Fig. 3 shows variations in DTP3 coverage trends from 2000 to 2022 by region and income group. Increasingly wide variation was seen within countries in Africa and LICs over time. Asia coverage shows some reversion to pre-pandemic coverage in 2022.

The combined model of income group and region explained a minimal amount of the variation in calculated deltas – adjusted R^2^ of the combined linear model for DTP3 was 20% in 2020, 14% 2021, and 6% in 2022. When conducting ANOVAs of the linear models of income group and region each year, heterogeneities due to one variable remained after accounting for the effect of the other in all but one permutation of variable order and year^3^ (details in **Appendix S9**).

### Country

**Fig. 4:**
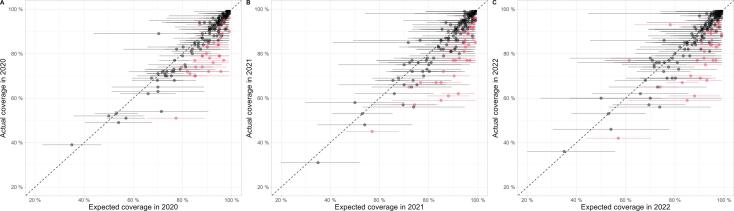
**Comparison between WUENIC-reported DTP3 coverage and expectations derived from historical trends:** This scatterplot shows country coverage (WUENIC-reported actuals and ARIMA-predicted expectations) as dots for 2020 (panel A), 2021 (panel B) and 2022 (panel C). Lines around individual points illustrate the 95% Confidence Intervals (CIs) of ARIMA predictions. Countries showing significant departure from expected values, *i.e.*, for which actual coverage is outside the 95% CI of predictions, are indicated in red; countries without such significant departure from expected results are shown in black. Countries with reported coverage lower than expected coverage are on the right-hand side of the dotted diagonal line. DTP3 = diphtheria, tetanus, and pertussis-containing vaccine third-dose. ARIMA = AutoRegressive Integrated Moving Average modelling. WUENIC = WHO/UNICEF Estimates of National Immunization Coverage. (For interpretation of the references to colour in this figure legend, the reader is referred to the web version of this article.)

The exact magnitude of country-level coverage changes remained hard to assess for many countries due to uncertainties in model predictions (Fig. 1), however evidence of coverage divergence from prior trends were significant for 25% of countries, and directional trends apparent for further countries. We plot individual country comparisons between expected and reported coverage each year in Fig. 4, with red dots indicating those countries with evidence of coverage being outside expected ranges. In 2020, 34 countries showed statistical evidence of having coverage outside expected ranges, increasing to 39 in 2021, and reducing to 33 in 2022 for DTP3. Of these datapoints all but two countries (Namibia in 2020, and Brazil and Iraq in 2022 - see **Appendix S11**) reported coverage below the expected levels.

**Fig. 5:**
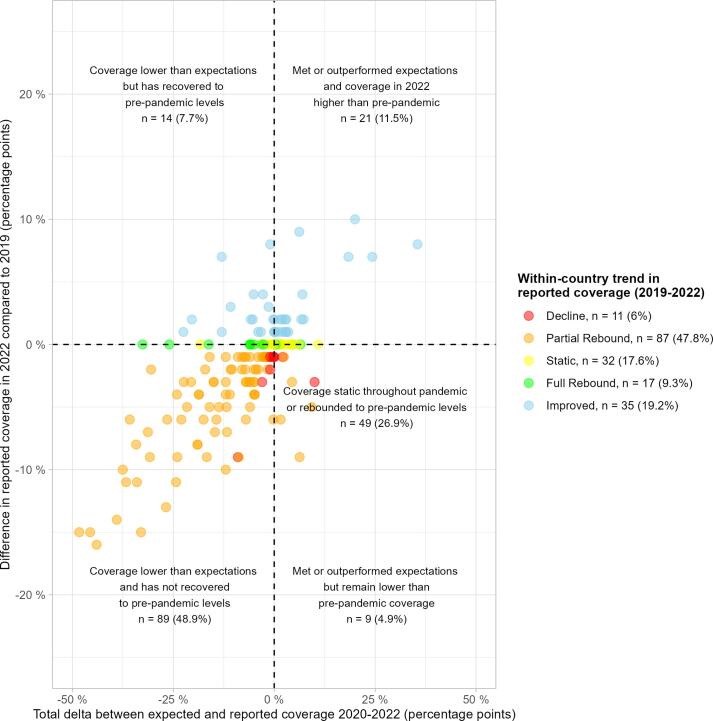
**Classification of pandemic impact and recovery by country for DTP3:** This scatterplot shows the combined 3-year (2020–2022) delta between expected and reported coverage on the x-axis and absolute difference between reported coverage in 2022 and 2019 on the y-axis. Labelling describes and quantifies (total and percentage of 182 countries) country classification with respect to the two plotted dimensions. DTP3 = diphtheria, tetanus, and pertussis-containing vaccine third-dose.

Fig. 5 illustrates the classification of country pandemic period immunisation performance in terms of absolute terms (*i.e.*, whether coverage increased or decreased in relation to 2019 levels) and relative trends (*i.e.*, whether coverage out- or under-performed compared to previous trajectories). For DTP3, 89 countries (48.9%) continue to report coverage lower than 2019 levels and lower than expected trends. Stagnation of coverage (either holding constant throughout the pandemic or reverting by 2022) is the second biggest trend, with 56 countries (26.9%). Only 21 countries (11.5%) have exceeded expected coverage and improved coverage to levels higher than 2019 – see ‘Discussion’ for details on insights and caveats for these high-performing countries.

### Missed immunisations

**Fig. 6:**
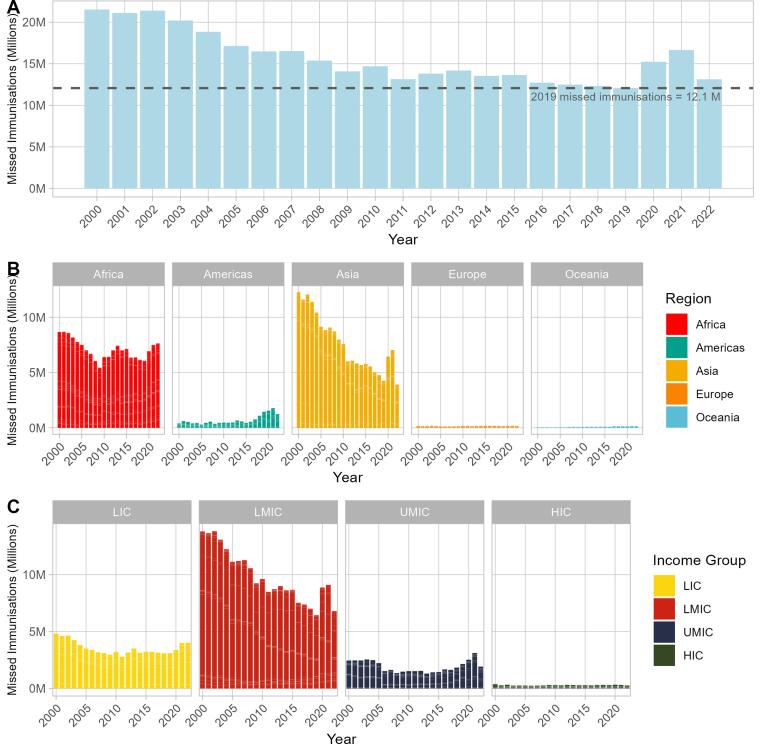
Number of Zero Dose (ZD) children per year from 2000 to 2022: panel A shows the global total with a dotted line indicating 2019 ZD levels; panel B breaks down the total by region and panel C by income group.

We focus on ZD children (DTP1-derived) estimates for this section, with estimates of children under-immunised based on DTP3 and MCV1 in **Appendix S12**. Pre-pandemic, in 2019 the number of ZD children in the 182 included countries was 12.1 M. Numbers of ZD had been relatively flat since 2016 (Fig. 6 **Panel A**), whilst declining in Asia (**Panel B**) and LMICs (**Panel C**) but increasing in the Americas (**Panel B**) and UMICs (**Panel C**).^4^ ZD children rose by almost 3 M in the first year of the pandemic, to 15.2 M in 2020, and further to 16.7 M in 2021, before falling to 13.1 M in 2022. The large improvement in 2022 was primarily driven by India, where DTP1 coverage increased 7 percentage points from 2021 to 2022 and the number of annual ZD children reduced by 1.6 M. In total during the pandemic period, compared to constant 2019 ZD levels this translates to an additional 8.8 M ZD children over three years.

The majority of additional ZD children during the pandemic were in Asia and LMICs (due to both population size and coverage deltas). In the Americas and UMICs ZD children continued to grow in 2020 and 2021 (continuing pre-pandemic trends) – but 2022 shows promising signs of reduced ZD populations.

At a country-level, Table 4 details the number of missed immunisations for the 10 countries with the absolute number of additional missed immunisations compared to 2019 levels.^5^ Of note, these countries are all in Asia or Africa and the majority are LICs or LMICs.

**Table 4:** Estimated Zero Dose (ZD) children for 10 countries with most additional ZD children total during 2020–2022 compared to 2019, calculated by DTP1 coverage deltas.

**Country**	**Region**	**Income group**	**Reported total ZD (2020**–**2022, M)**	**Additional ZD compared to 2019 levels (2020**–**2022, M)**	**Proportion additional (%)**
**India**	**Asia**	**LMIC**	**6.77**	**2.63**	**38.9**
**Indonesia**	**Asia**	**UMIC**	**2.48**	**1.13**	**45.8**
**Mozambique**	**Africa**	**LIC**	**0.94**	**0.64**	**68.8**
Nigeria	Africa	LMIC	6.73	0.63	9.3
**Ethiopia**	**Africa**	**LIC**	**3.17**	**0.54**	**16.9**
Angola	Africa	LMIC	1.66	0.44	26.5
**Philippines**	**Asia**	**LMIC**	**2.32**	**0.37**	**15.8**
Democratic Republic of the Congo	Africa	LIC	2.13	0.37	17.2
**United Republic of Tanzania**	**Africa**	**LMIC**	**0.85**	**0.27**	**31.4**
Cote d'Ivoire	Africa	LMIC	0.41	0.20	49.4

Caption: Proportion additional calculated as Additional Zero Dose (ZD) / Reported total ZD, where Additional ZD is calculated as the difference between WUENIC (reported) values and 2019 ZD numbers per country. Bold rows indicate countries where reported coverage was significantly lower than expected coverage for at least one year in 2020–2022. LIC: Low-income Country. LMIC: Lower-middle-income Country. UMIC: Upper-middle-income Country. HIC: High-income Country. DTP1 = diphtheria, tetanus, and pertussis-containing vaccine first-dose. WUENIC = WHO/UNICEF Estimates of National Immunization Coverage. M = million.

## Discussion

### Global RI coverage remains below pre-pandemic trends, with partial recovery in 2022

Our results provide further evidence that the global decline in RI coverage observed in 2020 continued in 2021 to a greater extent. We report DTP3 coverage decreasing from 89.7% in 2019 to a pandemic-period low of 86.4% in 2021, which was 3.4% [95% CIs: 2.5% − 4.4%] lower than expected based on previous trends. This aligns with recent publications [5], [6], [7] and is counter to indications of service delivery disruptions reducing at the time – WHO pulse surveys reported 61% of responding countries (n = 105) reported RI disruptions in health facilities in Q2 2020 [10], reducing to 34% (n = 89) in Q1 2021 [11] and 48% in Q4 2021 (n = 88) [9]. We report indications of tentative global RI recovery in 2022, with DTP3 coverage increasing by 0.8 percentage points from the prior year (when coverage was the lowest in 17-years) to revert to similar levels as seen in 2020 – *i.e.*, still remaining approximately 3% below pre-pandemic expectations (DTP3 2022 delta: −2.7% [-1.8%; −3.6%]). We believe this is the first estimate of the start of potential global pandemic recovery compared to expectations projected from pre-pandemic performance, with a considerable remaining coverage gap to address.

### Africa, the Americas, and Asia were hardest hit by pandemic RI disruptions

We show evidence of coverage declines in Africa, Asia, and the Americas in the first two years of the pandemic. Coverage in the Asia, and the Americas to a lesser extent, showed some recovery in 2022: Asia DTP3 deltas halved from 2021 to 2022 to be within the modelled confidence intervals (DTP3 delta: 1.6% [95% CI: −3.5%; 0.2%]) indicating some recovery to previous trends, but we note wide CIs in 2022 due to the limitations of projecting 2022 coverage from 2000 to 2019 trends. Coverage in Africa fell from 2019 to 2021 and remained relatively stagnate in 2022.

### HICs retained high coverage levels during the pandemic, whilst low- and middle-income countries exhibited declines versus expectations

Coverage trends varied by income group. LMICs exhibited curved trajectories of pandemic coverage declines, with peak impact in 2021; whilst UMICs coverage declines were greatest in 2020 and the gap to expected coverage reduced each year; and LICs impact increased from 2020 to 2021 and then only reduced slightly.

### Regional and income group variation indicates some areas for further exploration to understand pandemic impact

Variation in the timing and extent of pandemic impact and recovery between regions could be associated with differences in the epidemiology of COVID-19 across regions, and/ or by differences in pandemic response policies and interventions that could have affected health service access. Region and income group have independent explanatory power, but only explain 6–20% of variation in calculated deltas during 2020–2022, implying more variation in deltas remain to be explained. Further research is required to explore such associations and identify potential best practices for future pandemic response to enable maintenance and recovery of routine health services.

### RI continues to be at or below pre-pandemic levels in most countries globally

Based on absolute and relative classification of country-level coverage trends, RI performance has suffered or stagnated in 75.8% (138/ 182) of countries globally during the pandemic – considering DTP3:

• 48.9% of countries (89/182) report 2022 coverage remaining below pre-pandemic levels and below expected coverage – with the majority exhibiting partial rebounds in 2022 from pandemic lows
• 26.9% (49/ 182) stagnated at 2019 levels throughout the pandemic, or have fully rebounded coverage to 2019 levels in 2022 after coverage declines in 2020–2021

This indicates that immunisation services in most countries require renewed focus to fully recover to previous performance levels and improve coverage.

We hoped that the 21 countries (11.5%) that exceeded expected coverage and improved absolute coverage would provide success case studies and insight into factors for RI resilience during pandemics. However, caution is advised before interpretation in many of these countries – due to poor model fitting for some of these countries (*e.g*., Brazil) and a history of volatile immunisation coverage potentially due to small fluctuating birth cohorts (*e.g*., Marshall Islands, Samoa) meaning a track record of large coverage variations over time – see **Appendix S11**. Chad may be worth further exploration – DTP3 coverage grew year-on-year from 2015, including throughout the pandemic from 50% in 2019 to 60% in 2022.

More data and research at the sub-national level is required in order to determine the most appropriate catch-up routes and supplementary immunisation activities (SIAs), since sub-national heterogeneities in RI disruption likely underpin national estimates. WUENIC data is published at national level, therefore alternative coverage data sources, e.g., administration data (which has some known caveats – see **Appendix S1**) or other sub-national level surveys or studies would be required to provide this level of granularity (e.g., [35]).

### Catch-up is essential for the almost 9 M accumulated additional ZD children during the pandemic compared to 2019 levels

Notably, the number of annual ZD children in the 182 modelled countries increased by 4.6 M (38.0%) from 12.1 M 2019 to a peak of 16.7 M missed children in 2021. These results are in-line with WHO media releases [36], however point estimates are different, likely due to our exclusion of states lacking data or due to exclusion criteria. Time series analyses of number of missed immunisations could complement our research and allow deeper insights into where and how many un- and under-immunisations children there are.

The compounded impact of three years with RI coverage at declining or stagnated levels in many countries will place an increasing pressure on service delivery and supply chain – with a cumulative additional 8.8 M ZD from 2020 to 2022 to catch-up. Reaching children who missed immunisations during the pandemic (aged one, two, or three years old) places additional challenges on service delivery, as these children do not necessarily routinely interact with the immunisation system – immunisation platforms in the second year of life and beyond are highlighted as requiring further strengthening [37]. Recognising this, WHO, UNICEF, Gavi the Vaccine Alliance, and the Bill & Melinda Gates Foundation and broader partners launched “The Big Catch-up” [38] to “boost vaccination among children following declines driven by the COVID-19 pandemic”. Our research highlights how this, and related efforts, are important. Our ZD quantification could help (i) guide focus to countries with the greatest need based on additional ZD, and (ii) inform additional vaccine requirement planning, based on quantification of missed populations. In terms of greatest need, several individual countries with large birth cohorts reported coverage below expected, indicating evidence of large increases in ZD children during the pandemic. These countries include: India, Indonesia, Philippines and Ethiopia (see Table 3 above). These could be important focus countries for health system strengthening support.

The dropout between reported DTP1 and DTP3 coverage increased from 3.9% to 4.6% from 2019 to 2020 and then held constant for the following years. Comparing coverage deltas across the DTP immunisation schedule, the decline in DTP1 coverage was 63–76% of the size of the decline in DTP3 coverage each year. Together, this suggests that DTP3 coverage declines may have been driven ∼ 2/3rd by increases in ZD children (*i.e.*, lower DTP1 coverage) versus expectations, and ∼ 1/3rd by additional dropout between vaccine doses. On the one hand, this is particularly concerning given the risk of adverse health outcomes associated with ZD children [20] (e.g., disease outbreaks and VPD deaths). However, recognition that declines in coverage appear to be primarily driven by increases in ZD, rather than greater dropout between vaccine doses, provide insights on where to target limited resources for catch-up on health system strengthening. This places heightened importance on the increasing focus on channelling resources to identify and reach ZD children. Asia and the Americas showed strong (2 percentage points) improvements in DTP1 coverage from 2021 to 2022, which is encouraging and may provide a starting point for identifying best practices.

### Risk of more VPD outbreaks

Global measles coverage experienced a peak pandemic decline versus expectations in 2021 (MCV1 3.5% [95% CI: 2.4% − 4.6%] lower than expected; 85.6% expected coverage versus 89.1% expected). This is particularly concerning given the high sustained coverage (95%) required to prevent measles outbreaks [39]. Global disease surveillance suggests that coverage declines and/ or campaign postponements and delays are translating into an increase in worldwide measles outbreaks [40]. Modelling has shown that drops in RI coverage can result in increased VPD deaths [41] – leveraging such methods combined with inputting modelled pandemic coverage declines could help more precisely estimate potential increases in VPDs (though likely would also require sub-national datapoints). This could help proactively target campaigns where outbreaks could be most likely to occur.

### Factors for pandemic resilience

Whilst clear trends were seen across income group and regions, these two factors explain a small proportion of the global declines in coverage (adjusted R^2^ = 6–20% for the combined linear model, varying by year). Further understanding of explanatory factors is required to guide recovery and build resilience globally for future pandemics. Exploration of coverage changes with factors directly-associated with the pandemic (e.g., COVID-19 infection and mortality rates) and indirect factors (e.g., NPIs, and COVID-19 vaccine rollout) may be valuable starting points. Countries deployed a wide variety of NPIs over time, which influenced mobility and societal behaviours [42], and influenced immunisation services [43]. These interventions have been captured and codified in global datasets, such as the COVID-19 Government Response Tracker [44]. Exploring the relationship between NPI implementation and immunisation system performance over the last two years may indicate policy areas with beneficial and deleterious effects on health systems. Approximately 50% of countries surveyed by the WHO reported direct trade-offs between COVID-19 vaccination programme rollout and RI service delivery for infants and school-aged children [45]. Further quantification of the trade-off between COVID-19 vaccination and RI, in terms of morbidity and mortality implications, could be valuable in guiding immunisation strategies at the country- and global- level in the coming years as efforts to integrate COVID-19 in immunisation programmes continue.

## Limitations

Our results may under-estimate the extent of pandemic impact and recovery due to input data challenges and unreliable ARIMA-model fitting.

Where data is unavailable or not reported, WUENIC estimates hold coverage constant to the prior year – potentially obscuring actual declines or recoveries. This was true for 15 states in the latest release (*i.e.*, 2022 coverage held constant at 2021 coverage levels [24]). WUENIC also “retrospectively” revise previously-published coverage when new finalised data is reported by countries and deemed of sufficient quality. For this reason, we have updated ARIMA modelling for expected coverage in 2020 using the latest WUENIC data rather than leveraging previous results [19]. In addition, where reported administrative data is deemed of poor quality, WUENIC may calibrate to the latest available survey data levels. In such situations there is disconnect between country-reported and WUENIC-reported coverage, and the possibility that calibrated levels do not reflect the latest country immunisation context – or the full picture of pandemic impact and recovery. This is true for 44 countries (to varying extents) for DTP3 in the latest WUENIC data (see full WUENIC documentation [23], [24], [32]).

In addition, our results are limited to annual trends reported at a national level. Publicly available data on globally recognised estimates of coverage at a monthly level would enable more granular time series modelling on the impact of the pandemic on RI.

Finally, ARIMA model fitting may be skewed or unreliable where there has been large coverage changes or high volatility in coverage estimates, particularly for the years preceding 2019, as noted for Brazil where the ARIMA model projected out continued severe declines in immunisation based on a 20% decline in coverage from 2015 to 2019 (**Appendix S11**). We conducted exploratory sensitivity analyses where we conservatively removed such countries and found no impact on aggregate estimates.

## Conclusion

This research builds on our previously published, transparent, and replicable approach for estimating gaps in RI coverage across countries, providing an objective measure for missed immunisations and coverage disruptions each year. Indications of a partial rebound in 2022 are promising, however global coverage remains at 2005 levels and immunisation services continue to be disrupted in Africa, Asia, and the Americas. The compound impact of the pandemic over three years is associated with a build-up of almost 9 M additional ZD children to catch-up. We hope this work can inform future research to identify effective interventions to facilitate rebounds in coverage to previous levels and catch-up the cohorts of under- and un-immunised children.

## Declaration of Competing Interest

The authors declare the following financial interests/personal relationships which may be considered as potential competing interests: It is noted that BE has been employed by the Clinton Health Access Initiative in the Global Vaccines team in the last three years; and is currently employed by Gavi, the Vaccine Alliance. All research contained in this manuscript was conducted during a doctorate qualification, outside and independent of employment. Neither facilities, data, nor any other forms of input from the Clinton Health Access Initiative or Gavi, were used in this study. The research and manuscript are independent of the Clinton Health Access Initiative and Gavi, and the findings have not been discussed, reviewed, or endorsed by the Clinton Health Access Initiative, the Gavi Secretariat, or any Alliance members.

## Footnotes

1 based on current surviving infant cohorts and coverage levels [23], [25].

2 American Samoa, Belize, El Salvador, Guinea, Guyana, Haiti, Indonesia, Iran, Jordan, Lebanon, Mauritius, Moldova, Palau, Panama, Romania, Samoa, Tajikistan, West Bank and Gaza, and Zambia.

3 Excluding instance: *p-*value of 0.07 for the ANOVA quantifying the effect of region after income group in 2022.

4 Driven by ∼20% declines in DTP1 coverage in large countries from > 95% coverage to ∼80% in Brazil, Mexico, and Argentina since 2015.

5 Noting Table 3 is ordered by ‘Additional ZD (2020–2022, M) compared to 2019 levels. This means that countries with large ZD populations, but high ‘baseline’ ZD in 2019, e.g., Brazil, Argentina, and Mexico, are not included.
