# Supplementary material for: Analysis of global routine immunisation coverage shows disruption and stagnation during the first two-years of the COVID-19 pandemic with tentative recovery in 2022
Source: Vaccine X. 2023 Sep 6;15:100383. doi: 10.1016/j.jvacx.2023.100383 (PMC10568411; doi:10.1016/j.jvacx.2023.100383)
Supplement: Supplementary data 1 [file mmc1.docx]

**Supplementary Material: Additional data, methodological information, and complementary results**

**Captions:**

- Supplementary material (Word file): This supplementary text provides further details on: selection of datasets, methodologies used for modelling, countries/ datapoints removed from analyses. It also provides full outputs across all countries, and links to the GitHub repository for original datasets and R code.
- Supplementary Tables (Excel file): This table includes results for all countries for DTP1, DTP3, and MCV1 modelling for 2020, 2021, and 2022 in an accessible Excel format. There is one tab for each vaccine dose, and details in the Word document on how to interpret.

**Contents:**

Section S1: Vaccination coverage data selection

Section S2: Further datasets for categorising countries

Section S3: Using ARIMA to model expected vaccination coverage

Section S4: Countries removed from analyses

Section S5: Full country-level results for 2020-2022

Section S6: Global results for 2020-2022

Section S7: Consolidated region results for 2020-2022

Section S8: Consolidated income group results for 2020-2022

Section S9: Income and region interaction results

Section S10: Country analyses

Section S11: Countries classified as improved immunisation performance

Section S12: Missed immunisations

Section S13: Availability and reproducibility

**Section S1: Vaccination coverage data selection**

Vaccine coverage is typically estimated through (a) aggregating (raw) administration data, or (b) conducting surveys. Expert opinion and/ or statistical methods may be layered on to produce final estimates. Administration data is the most timely and periodic, but risks numerator (e.g., under- or over-estimation dependent on health system capacity, reporting incentives, and linkage to private sector systems) and denominator (e.g., out-of-date censuses used for population quantification) biases [1]. Surveys, typically household or parental, avoid such biases but are expensive, time-consuming, infrequent, and may encounter recall bias, i.e., parents or guardians may mis-remember or confuse vaccination statuses when reporting due to complex immunisation schedules and potentially long periods between delivery and surveys [2]. The Institute for Health Metrics and Evaluation (IHME) produces coverage estimates by applying spatiotemporal statistical methods to household survey microdata (if available) and estimates of country-reported coverage data [3]. IHME usefully publish confidence ranges, unlike other sources, but methods are not fully reproducible nor used routinely by global immunisation stakeholders to assess immunisation performance. WUENIC estimates are published annually through computational logic rule-based approaches that use inputs from country-reported administrative and survey data, adjusted based on expert assessment and country consultations [4], [5]. WUENIC estimates are transparent, replicable, and routinely used by stakeholders to inform policy, financial, and programmatic decisions.

WUENIC data was selected due to its transparency, replicability, utility by key organisations and donors working on or investing in immunisation globally (e.g., UNICEF, WHO and Gavi), and public availability. From this data, three RIs – DTP1, DTP3 and MCV1 – were selected since they act as key immunisation indicators.

**Section S2: Further datasets for categorising countries**

Three additional sources were used to assemble country demographic information – population data from the United Nations World Population Prospects (UNWPP [6]), World Bank (WB) income group classification [7], and United Nations (UN) regional classifications (using the “countrycode” package in R, [8]).

**Section S3: Using ARIMA to model expected vaccination coverage**

WUENIC does not publish future-looking coverage forecasts. Expected coverage for 2020 and 2021, by country in the absence of COVID-19 was modelled by fitting AutoRegressive Integrated Moving Average (ARIMA) models to 20-years of annual WUENIC coverage data (2000-2019) using the package “*forecast*”) in R [9] and projecting models forward for two years. No projections were modelled for countries missing any coverage values from 2000 to 2019.

A description of how ARIMA forecasting selects models is included in the ‘Methods and materials’ section of the manuscript.

The mean ARIMA-modelled value was selected per country and vaccine as expected 2020 coverage. WUENIC cap coverage at 99% [4], [5]. To avoid falsely calculating small declines in coverage for countries where ARIMA-predicted 2020 coverage was higher than 99%, all expected coverage estimates were capped at the WUENIC maximum.

**Section S4: Countries removed from analyses**

Before further analysis, coverage time series including expected coverage were investigated to identify unreliable estimates as described in the manuscript.

The following countries were removed from all analyses:

- Countries lacking recent WUENIC coverage updates: For five countries (the Central African Republic, Haiti, Guinea, Lesotho, and Somalia), WUENIC report an inability to update coverage estimates for multiple years due to the absence of additional information meeting their criteria, resulting in WUENIC coverage estimates appearing as constant.
- Countries where major non-COVID-specific events have reportedly affected immunisation: This applied to Myanmar, Ukraine, and the Democratic People’s Republic of Korea where immunisation coverage reportedly fell catastrophically in recent years. Whilst some of these coverage declines may have been associated with the pandemic, to be conservative we removed these countries for aggregate analyses.

Coverage trends from 2000-2022 and modelled ARIMA expected coverage for 2020, 2021 and 2022, and associated 95% confidence intervals, for each removed country can be seen in Figure S4.1 for DTP1, S4.2 for DTP3, and S4.3 for MCV1.


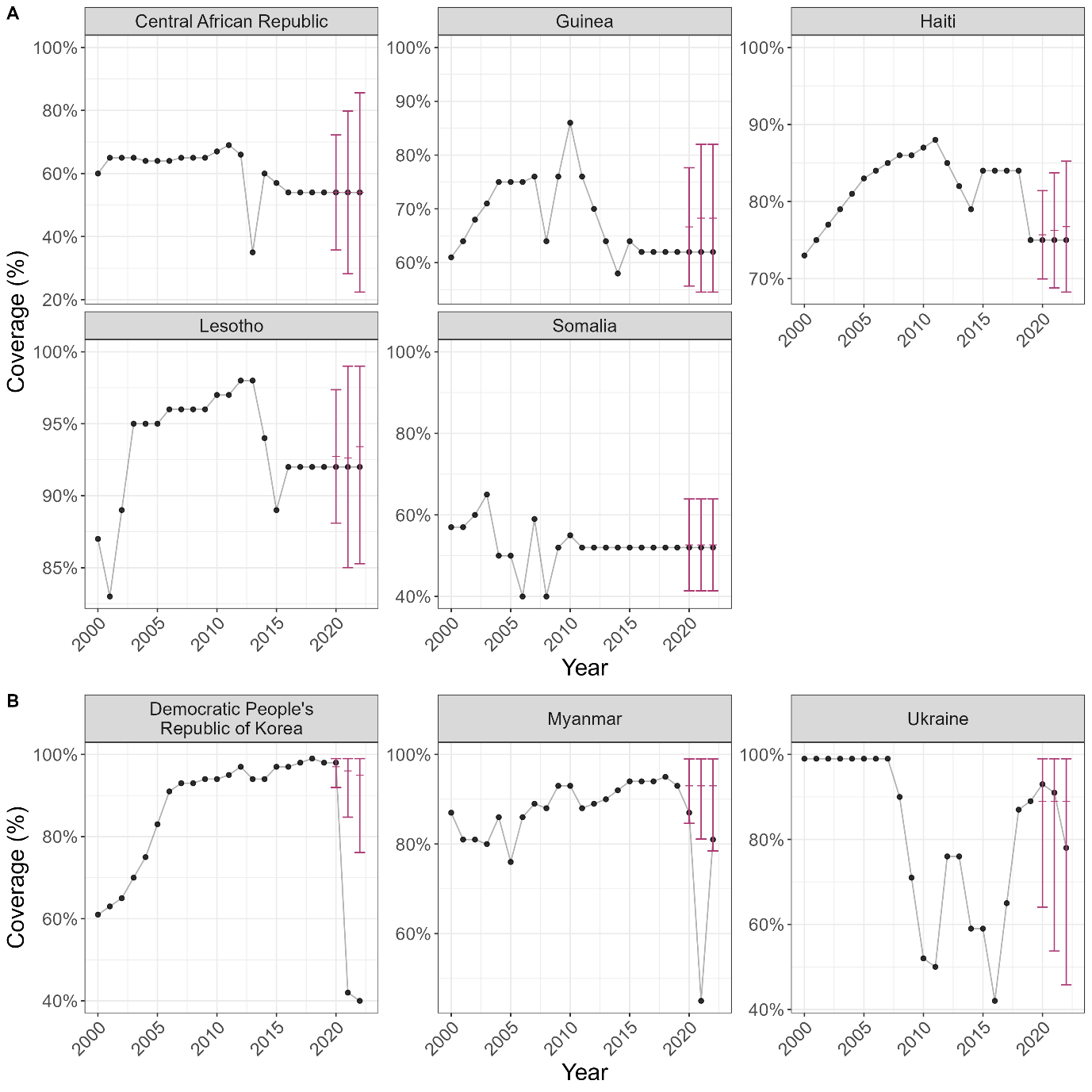


**Figure S4.1: Expected and reported 2020-20212 vaccine coverage for DTP1 for countries removed from aggregate analyses.** These graphs show WUENIC-reported coverage data (black dots) from 2000 to 2022 inclusive, and the corresponding ARIMA predictions and the associated 95% CIs (red bars) for 2020 and 2022. Panel A shows countries removed due to lack of up-to-date WUENIC estimates, and Panel B shows countries with large where major geopolitical events are reported to have affected routine immunisation.


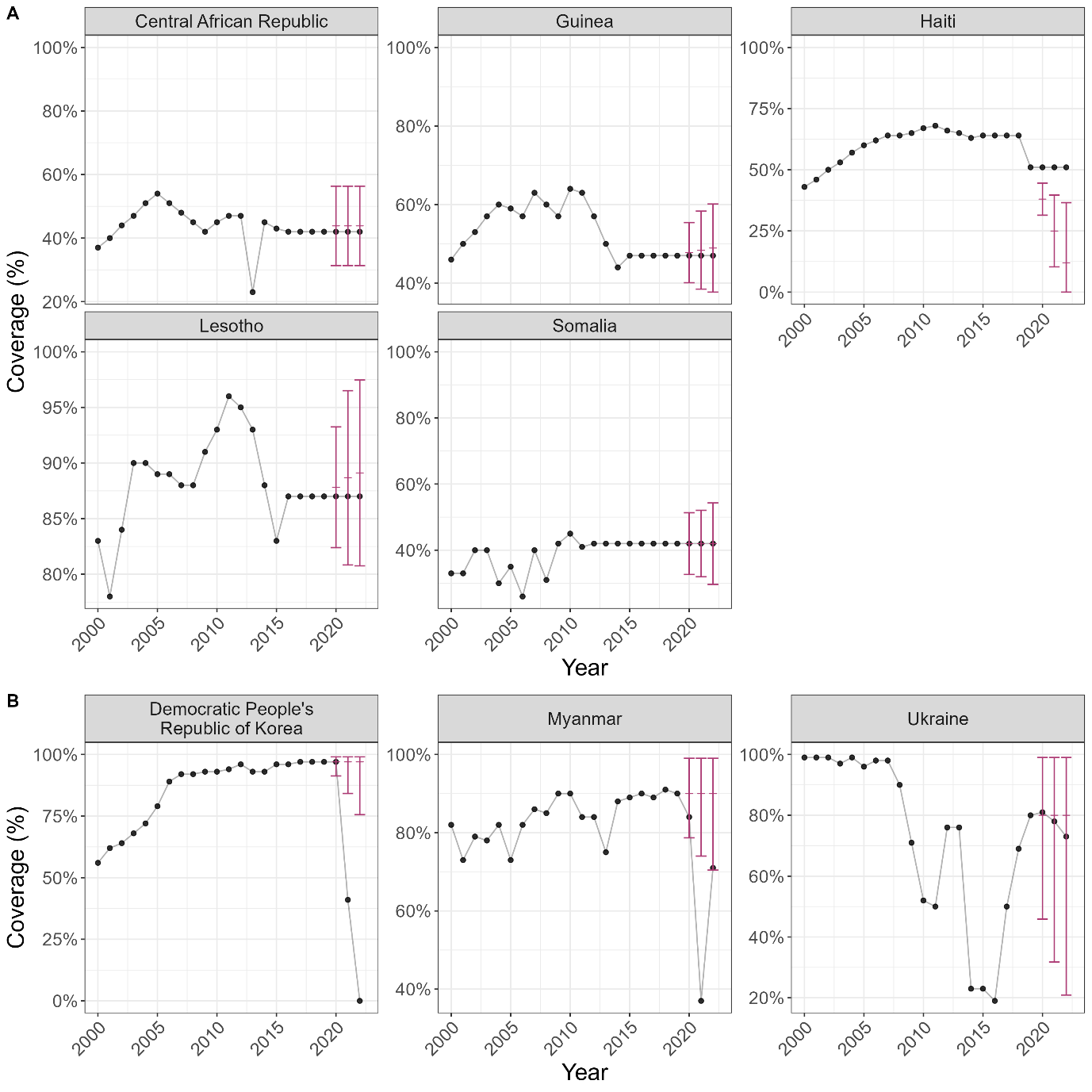


**Figure S4.2: Expected and reported 2020-20212 vaccine coverage for DTP3 for countries removed from aggregate analyses.** These graphs show WUENIC-reported coverage data (black dots) from 2000 to 2022 inclusive, and the corresponding ARIMA predictions and the associated 95% CIs (red bars) for 2020 and 2022. Panel A shows countries removed due to lack of up-to-date WUENIC estimates, and Panel B shows countries with large where major geopolitical events are reported to have affected routine immunisation.


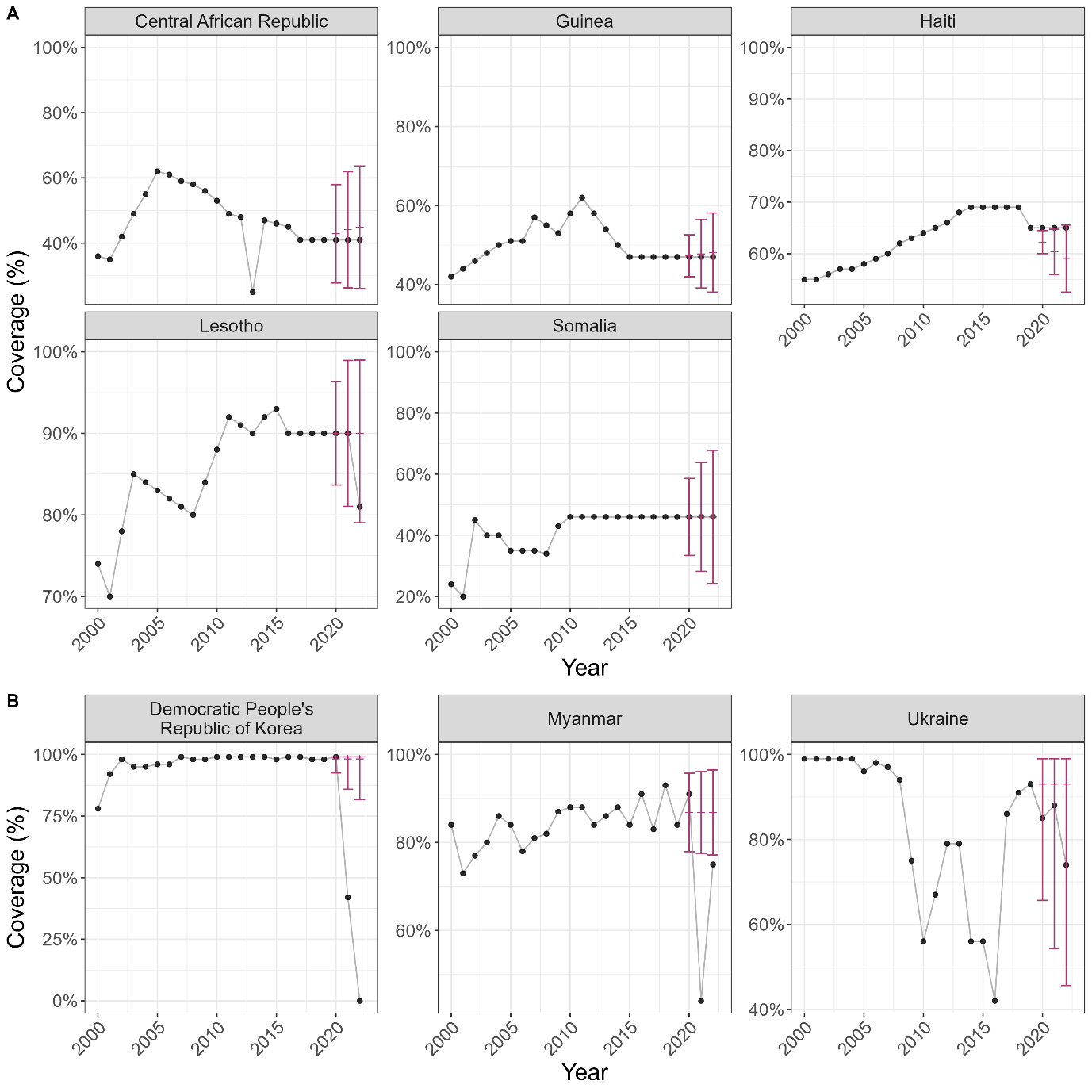


**Figure S4.3: Expected and reported 2020-20212 vaccine coverage for MCV1 for countries removed from aggregate analyses.** These graphs show WUENIC-reported coverage data (black dots) from 2000 to 2022 inclusive, and the corresponding ARIMA predictions and the associated 95% CIs (red bars) for 2020 and 2022. Panel A shows countries removed due to lack of up-to-date WUENIC estimates, and Panel B shows countries with large where major geopolitical events are reported to have affected routine immunisation.

**Section S5: Full country-level results for 2020 and 2021**

Full output results in a file format from which data can be efficiently extracted are available in **Supplementary Materials Table S1** for DTP1, and **Supplementary Materials Table S2** for DTP3, and **Supplementary Materials Tables S3** for MCV1. These tables include results for 2020, 2021, and 2022:

- Country classification details: country, ISO code, UN region and income group
- ARIMA predictions: ARIMA-forecast ‘expected’ coverage, low- and high- 95% confidence intervals
- WUENIC reported coverage
- Deltas (expected - reported coverage): mean, low- and high- 95% confidence intervals
- 95% confident flag: binary flag whether WUENIC-reported 2020 coverage is outside the ARIMA-predicted 95% confidence intervals (CIs) each year. TRUE if within CIs, and FALSE if outside CIs.
- Surviving infant population: from UN WPP 2022
- Missed immunisation estimates: total missed immunisations (based on WUENIC-reported coverage), Expected (based on forecast ARIMA prediction coverage) for each year

**Section S6: Global results for 2020-2022**

In all tables, ‘expected coverage’ is used as in the manuscript to reflect ARIMA-projected coverage levels based on 2000-2019 time series, and ‘reported coverage’ details WUENIC-reported coverage. *p*-values reflect results from *t*-tests.

*S6(a) T-test of global impact – final results, DTP1*

| **Year** | **Expected coverage** | **Reported coverage** | **Delta [95% Confidence Intervals]** | ***p-*value** | **Coverage last seen in** |
| --- | --- | --- | --- | --- | --- |
| 2020 | 93.6% | 91.6% | -2.0% [-1.3%; -2.7%] | < 0.0001 | 2004 |
| 2021 | 93.6% | 91.0% | -2.6% [-1.8%; -3.4%] | < 0.0001 | 2002 |
| 2022 | 93.6% | 91.8% | -1.7% [-0.8%; -2.6%] | 0.00026 | 2004 |

*S6(b) T-test of global impact – final results, DTP3*

Reported in manuscript as **Table 1**

*S6(c) T-test of global impact – final results, MCV1*

| **Year** | **Expected coverage** | **Reported coverage** | **Delta [95% Confidence Intervals]** | ***p-*value** | **Coverage last seen in** |
| --- | --- | --- | --- | --- | --- |
| 2020 | 88.9% | 85.9% | -3.0% [-2.2%; -3.8%] | < 0.0001 | 2005 |
| 2021 | 88.9% | 84.7% | -4.2% [-3.2%; -5.3%] | < 0.0001 | 2004 |
| 2022 | 89.1% | 85.6% | -3.5% [-2.4%; -4.6%] | < 0.0001 | 2005 |

**Section S7: Consolidated region results for 2020-2022**

Table details output from conducting a t-test on the difference between ARIMA-forecasted (expected) and WUENIC-reported (reported) coverage each year by region for DTP3. Deltas are the difference between expected and reported coverage, with 95% Confidence Intervals (CIs) in square brackets. Negative deltas mean coverage was lower than expected. Bold rows indicate where reported coverage is outside (lower, in all cases) the expected 95% CIs.

*S7(a) T-tests by region – DTP1*

1. *DTP1 2020*

| **Region** | **Sample size** | **Expected coverage** | **Reported coverage** | **Delta [95% Confidence Intervals)** | **p-value** |
| --- | --- | --- | --- | --- | --- |
| **Africa** | **49** | **89.8%** | **87.2%** | **-2.6% [-4.0%; -1.3%]** | **0.00013** |
| **Americas** | **34** | **93.3%** | **89.4%** | **-3.9% [-5.5%; -2.3%]** | **< 0.0001** |
| **Asia** | **45** | **95.3%** | **92.9%** | **-2.4% [-3.8%; -1.0%]** | **0.00078** |
| Europe | 40 | 97.2% | 96.6% | -0.6% [-2%; 0.9%] | 0.46 |
| Oceania | 14 | 92.0% | 93.6% | 1.6% [-0.9%; 4.0%] | 0.22 |

1. *DTP1 2021*

| **Region** | **Sample size** | **Expected coverage** | **Reported coverage** | **Delta [95% Confidence Intervals)** | **p-value** |
| --- | --- | --- | --- | --- | --- |
| **Africa** | **49** | **90.0%** | **86.6%** | **-3.4% [-5.0%; -1.8%]** | **< 0.0001** |
| **Americas** | **34** | **93.2%** | **89.0%** | **-4.1% [-6.0%; -2.2%]** | **< 0.0001** |
| **Asia** | **45** | **95.1%** | **92.6%** | **-2.6% [-4.2%; -0.9%]** | **0.0027** |
| Europe | 40 | 97.2% | 96.2% | -1.0% [-2.8%; 0.8%] | 0.26 |
| Oceania | 14 | 91.7% | 91.2% | -0.5% [-3.5%; 2.5%] | 0.73 |

1. *DTP1 2022*

| **Region** | **Sample size** | **Expected coverage** | **Reported coverage** | **Delta [95% Confidence Intervals)** | **p-value** |
| --- | --- | --- | --- | --- | --- |
| **Africa** | **49** | **90.1%** | **86.7%** | **-3.4% [-5.1%; -1.6%]** | **0.00019** |
| Americas | 34 | 92.8% | 90.9% | -2.0% [-4.1%; 0.1%] | 0.064 |
| Asia | 45 | 95.2% | 94.2% | -1.0% [-2.8%; 0.8%] | 0.27 |
| Europe | 40 | 97.2% | 96.0% | -1.2% [-3.1%; 0.8%] | 0.24 |
| Oceania | 14 | 91.7% | 92.5% | 0.8% [-2.5%; 4.1%] | 0.64 |

*S7(b) T-tests by region – DTP3*

Reported in the manuscript as **Table 2**

*S7(c) T-tests by region – MCV1*

1. *MCV1 2020*

| **Region** | **Sample size** | **Expected coverage** | **Reported coverage** | **Delta [95% Confidence Intervals)** | **p-value** |
| --- | --- | --- | --- | --- | --- |
| **Africa** | **49** | **80.7%** | **77.9%** | **-2.8% [-4.3%; -1.3%]** | **0.00039** |
| **Americas** | **34** | **92.2%** | **85.9%** | **-6.3% [-8.1%; -4.5%]** | **< 0.0001** |
| **Asia** | **45** | **92.7%** | **90.0%** | **-2.7% [-4.3%; -1.1%]** | **0.0011** |
| Europe | 40 | 93.3% | 91.6% | -1.7% [-3.3%; 0.0%] | 0.056 |
| Oceania | 14 | 85.2% | 84.6% | -0.6% [-3.4%; 2.3%] | 0.7 |

1. *MCV1 2021*

| **Region** | **Sample size** | **Expected coverage** | **Reported coverage** | **Delta [95% Confidence Intervals)** | **p-value** |
| --- | --- | --- | --- | --- | --- |
| **Africa** | **49** | **81.1%** | **77.0%** | **-4.0% [-6.1%; -2.0%]** | **0.00013** |
| **Americas** | **34** | **92.0%** | **83.7%** | **-8.3% [-10.7%; -5.8%]** | **< 0.0001** |
| **Asia** | **45** | **92.8%** | **89.6%** | **-3.2% [-5.3%; -1.1%]** | **0.0035** |
| Europe | 40 | 93.3% | 91.1% | -2.2% [-4.5%; 0.0%] | 0.054 |
| Oceania | 14 | 84.0% | 79.4% | -4.6% [-8.4%; -0.8%] | 0.019 |

1. *MCV1 2022*

| **Region** | **Sample size** | **Expected coverage** | **Reported coverage** | **Delta [95% Confidence Intervals)** | **p-value** |
| --- | --- | --- | --- | --- | --- |
| **Africa** | **49** | **81.3%** | **77.6%** | **-3.7% [-5.7%; -1.7%]** | **0.00042** |
| **Americas** | **34** | **92.5%** | **84.4%** | **-8.2% [-10.6%; -5.7%]** | **< 0.0001** |
| **Asia** | **45** | **92.8%** | **90.6%** | **-2.3% [-4.4%; -0.1%]** | **0.036** |
| **Europe** | **40** | **93.3%** | **90.8%** | **-2.5% [-4.7%; -0.3%]** | **0.029** |
| Oceania | 14 | 84.0% | 84.9% | 0.9% [-2.8%; 4.7%] | 0.63 |

**Section S8: Consolidated income group results for 2020-2022**

*S8(a) T-tests by income group – DTP1*

1. *DTP1 2020*

| **Income group** | **Sample size** | **Reported coverage** | **Expected coverage** | **Delta** | **p-value** |
| --- | --- | --- | --- | --- | --- |
| **LIC** | **22** | **85.7%** | **87.9%** | **-2.2% [-4.2%; -0.2%]** | **0.03** |
| **LMIC** | **48** | **88.6%** | **91.6%** | **-3.0% [-4.4%; -1.7%]** | **< 0.0001** |
| **UMIC** | **52** | **90.9%** | **93.6%** | **-2.8% [-4.0%; -1.5%]** | **< 0.0001** |
| HIC | 59 | 97.2% | 97.5% | -0.3% [-1.5%; 0.9%] | 0.62 |

1. *DTP1 2021*

| **Income group** | **Sample size** | **Reported coverage** | **Expected coverage** | **Delta** | **p-value** |
| --- | --- | --- | --- | --- | --- |
| **LIC** | **22** | **83.8%** | **87.9%** | **-4.1% [-6.4%; -1.8%]** | **0.00051** |
| **LMIC** | **48** | **87.1%** | **91.6%** | **-4.5% [-6.1%; -2.9%]** | **< 0.0001** |
| **UMIC** | **52** | **91.2%** | **93.4%** | **-2.3% [-3.8%; -0.8%]** | **0.0029** |
| HIC | 59 | 96.9% | 97.4% | -0.5% [-1.9%; 0.9%] | 0.47 |

1. *DTP1 2022*

| **Income group** | **Sample size** | **Reported coverage** | **Expected coverage** | **Delta** | **p-value** |
| --- | --- | --- | --- | --- | --- |
| **LIC** | **22** | **84.6%** | **88.1%** | **-3.4% [-6.0%; -0.9%]** | **0.0094** |
| **LMIC** | **48** | **88.7%** | **91.7%** | **-3.0% [-4.7%; -1.2%]** | **0.00093** |
| UMIC | 52 | 92.2% | 93.3% | -1.1% [-2.8%; 0.6%] | 0.19 |
| HIC | 59 | 97.1% | 97.4% | -0.4% [-2.0%; 1.2%] | 0.63 |

*S7(b) T-tests by income group – DTP3:* Reported in the manuscript as **Table 3**

*S7(c) T-tests by income group – MCV1*

1. *MCV1 2020*

| **Income group** | **Sample size** | **Reported coverage** | **Expected coverage** | **Delta** | **p-value** |
| --- | --- | --- | --- | --- | --- |
| **LIC** | **22** | **74.5%** | **77.6%** | **-3.1% [-5.4%; -0.8%]** | **0.0093** |
| **LMIC** | **48** | **82.1%** | **85.5%** | **-3.4% [-4.9%; -1.8%]** | **< 0.0001** |
| **UMIC** | **52** | **85.5%** | **90.4%** | **-4.9% [-6.4%; -3.4%]** | **< 0.0001** |
| HIC | 59 | 93.8% | 94.8% | -0.9% [-2.3%; 0.5%] | 0.19 |

1. *MCV1 2021*

| **Income group** | **Sample size** | **Reported coverage** | **Expected coverage** | **Delta** | **p-value** |
| --- | --- | --- | --- | --- | --- |
| **LIC** | **22** | **73.2%** | **78.1%** | **-4.9% [-7.9%; -1.9%]** | **0.0015** |
| **LMIC** | **48** | **79.0%** | **85.5%** | **-6.5% [-8.5%; -4.5%]** | **< 0.0001** |
| **UMIC** | **52** | **85.3%** | **90.1%** | **-4.9% [-6.8%; -2.9%]** | **< 0.0001** |
| HIC | 59 | 93.4% | 94.7% | -1.4% [-3.2%; 0.4%] | 0.14 |

1. *MCV1 2022*

| **Income group** | **Sample size** | **Reported coverage** | **Expected coverage** | **Delta** | **p-value** |
| --- | --- | --- | --- | --- | --- |
| **LIC** | **22** | **73.7%** | **78.5%** | **-4.8% [-7.9%; -1.7%]** | **0.0024** |
| **LMIC** | **48** | **81.4%** | **85.5%** | **-4.1% [-6.2%; -2%]** | **0.00014** |
| **UMIC** | **52** | **85.6%** | **90.5%** | **-4.9% [-6.9%; -2.9%]** | **< 0.0001** |
| HIC | 59 | 93.6% | 94.8% | -1.2% [-3.1%; 0.7%] | 0.21 |

**Section 9: Region and income group interaction – DTP3**

| **Year** | **Degrees of freedom (df)** | **Region after income** | | **Income after region** | |
| --- | --- | --- | --- | --- | --- |
|  |  | ***F-*value** | ***p-value*** | ***F-*value** | ***p-value*** |
| 2020 | 173 | **9.16** | **< 0.0001** | **3.22** | **0.02** |
| 2021 | 173 | **4.58** | **0.002** | **6.60** | **0.0003** |
| 2022 | 173 | 2.22 | 0.07 | **2.86** | **0.04** |

*Caption:* Table of F- and p-values from ANOVAs conducted on the combined linear model of region and income group with coverage changes as the response variable for DTP3. Bold rows indicate where heterogeneities due to one explanatory factor remained after accounting for the effect of the other (*p* < 0.05).

**Section S10: Country analyses**

*S10(a) Comparison between WUENIC-reported DTP3 coverage and expectations derived from historical trends – recreates Figure 4 of the manuscript for the other vaccine doses per year*

1. *DTP1 2020*


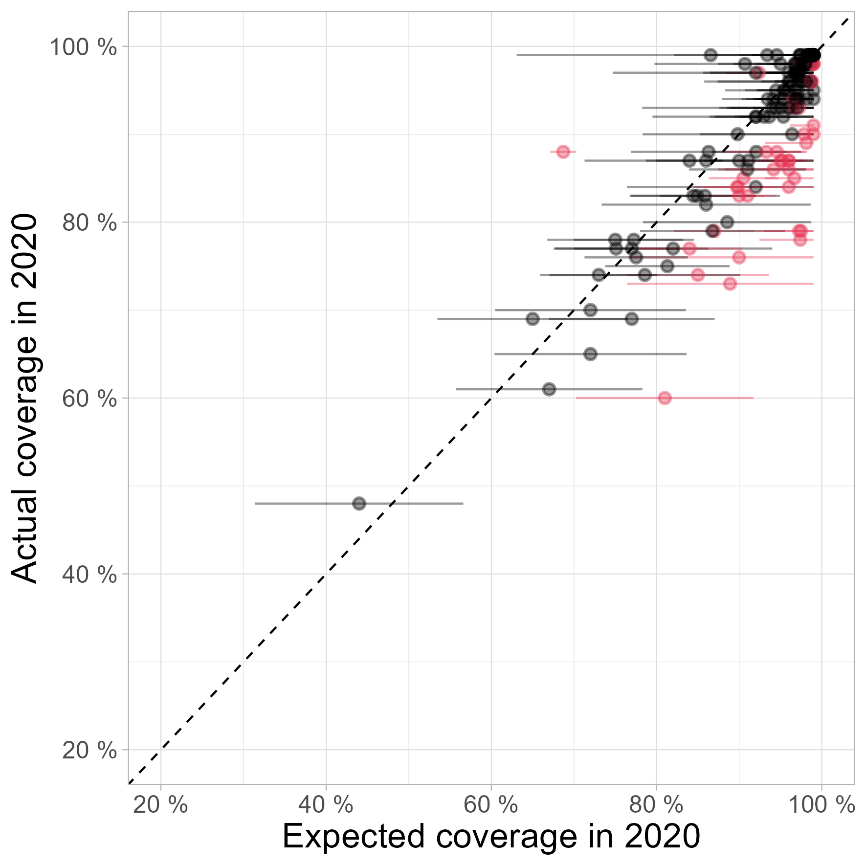


1. *DTP1 2021*

*
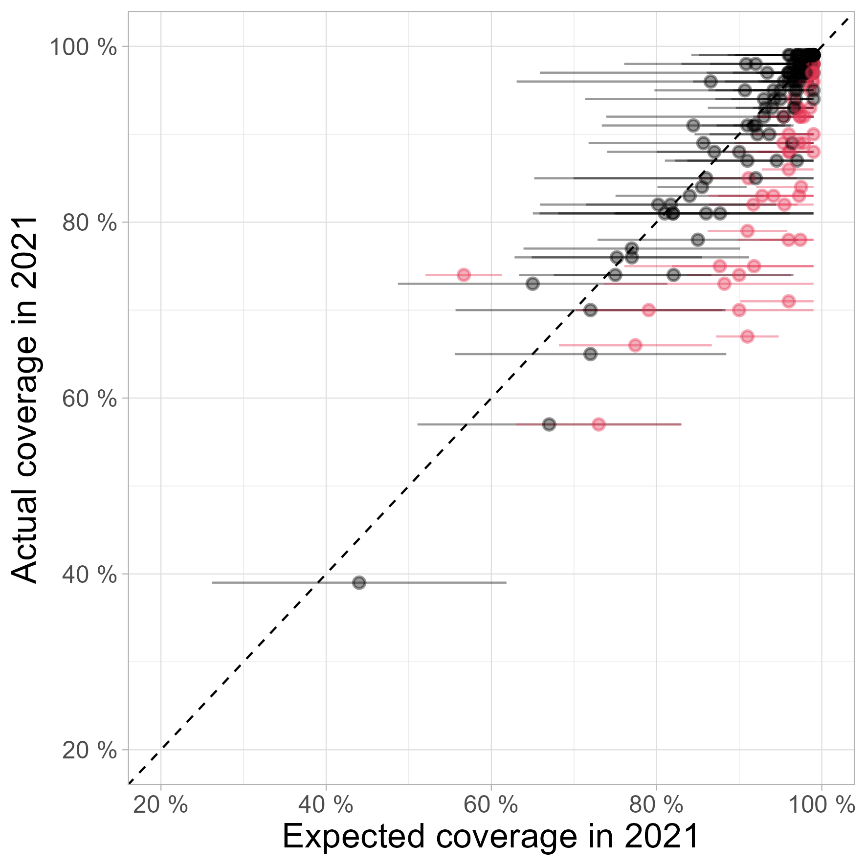
*

1. *DTP1 2022*

*
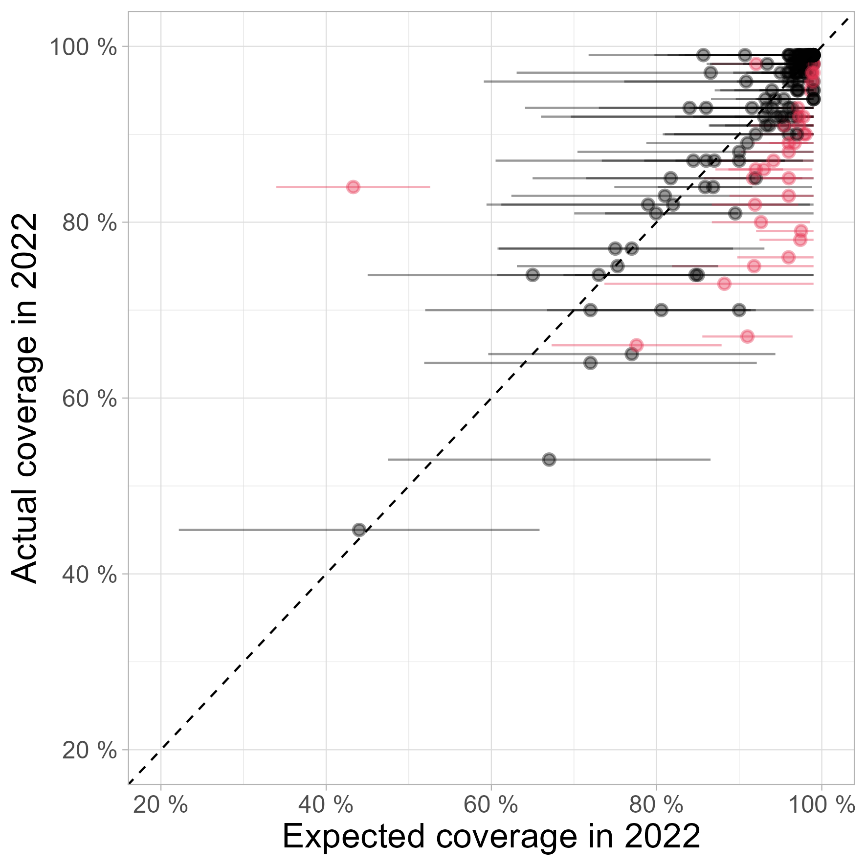
*

1. *MCV1 2020*

*
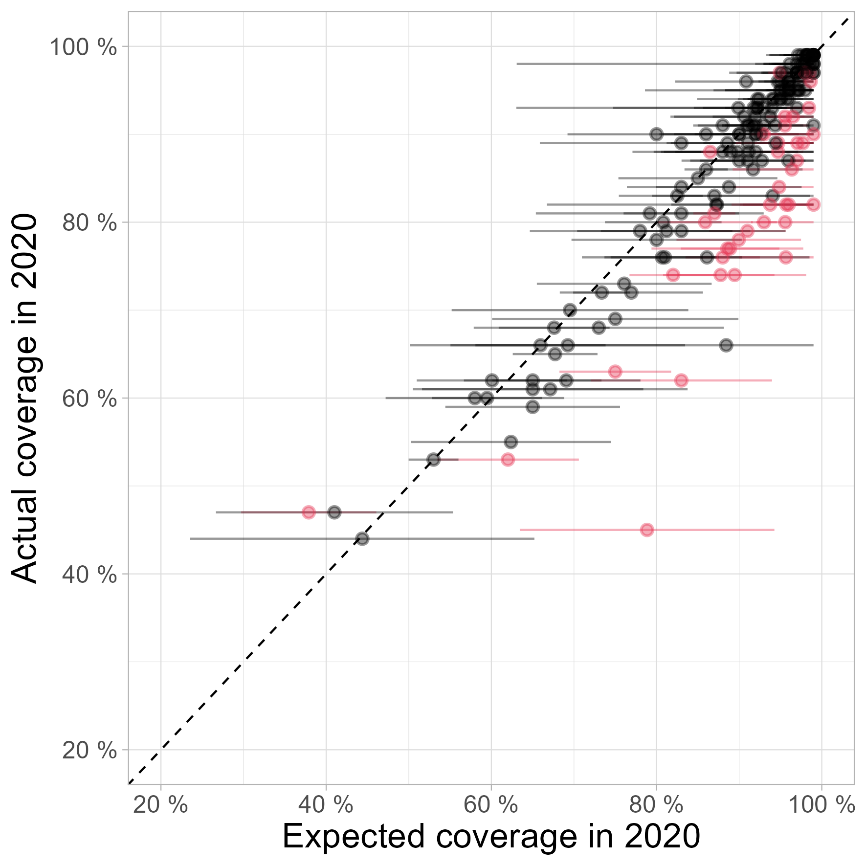
*

1. *MCV1 2021

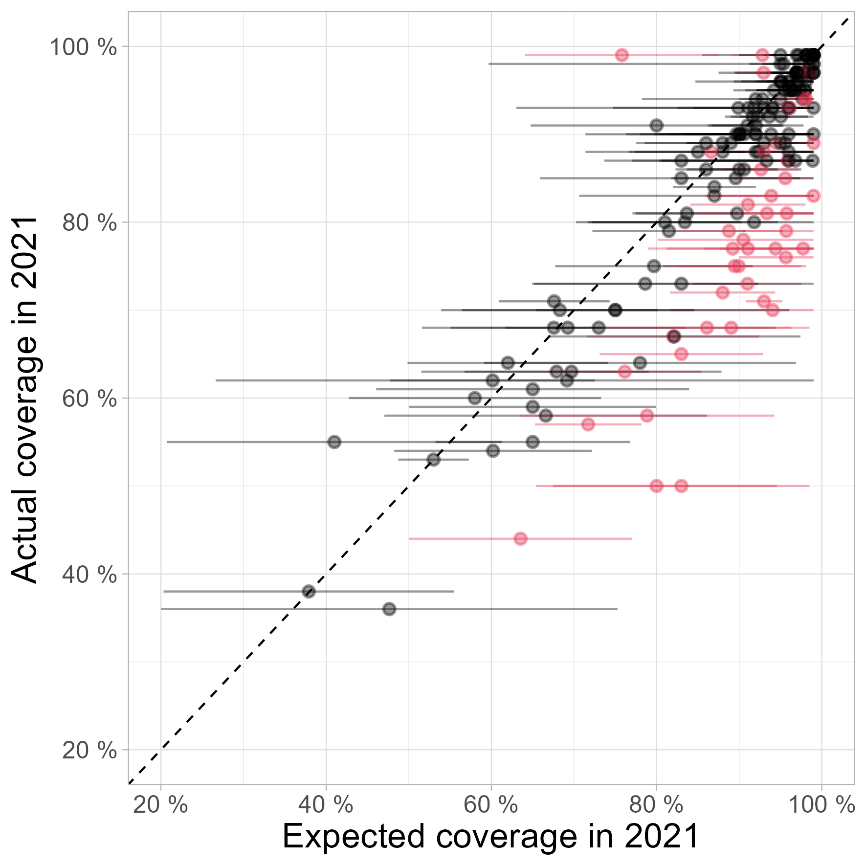
*
2. *MCV1 2022*

*
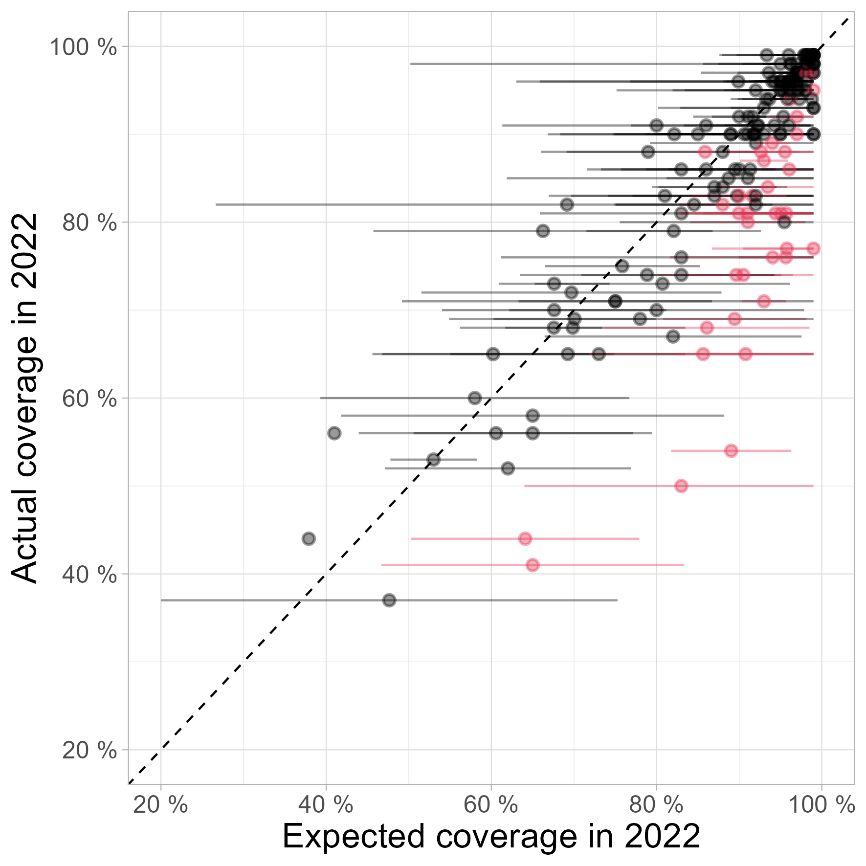
*

*S10(b) Classification of pandemic impact and recovery by country – recreates Figure 5 of the manuscript for the other vaccine doses*

1. *DTP1*


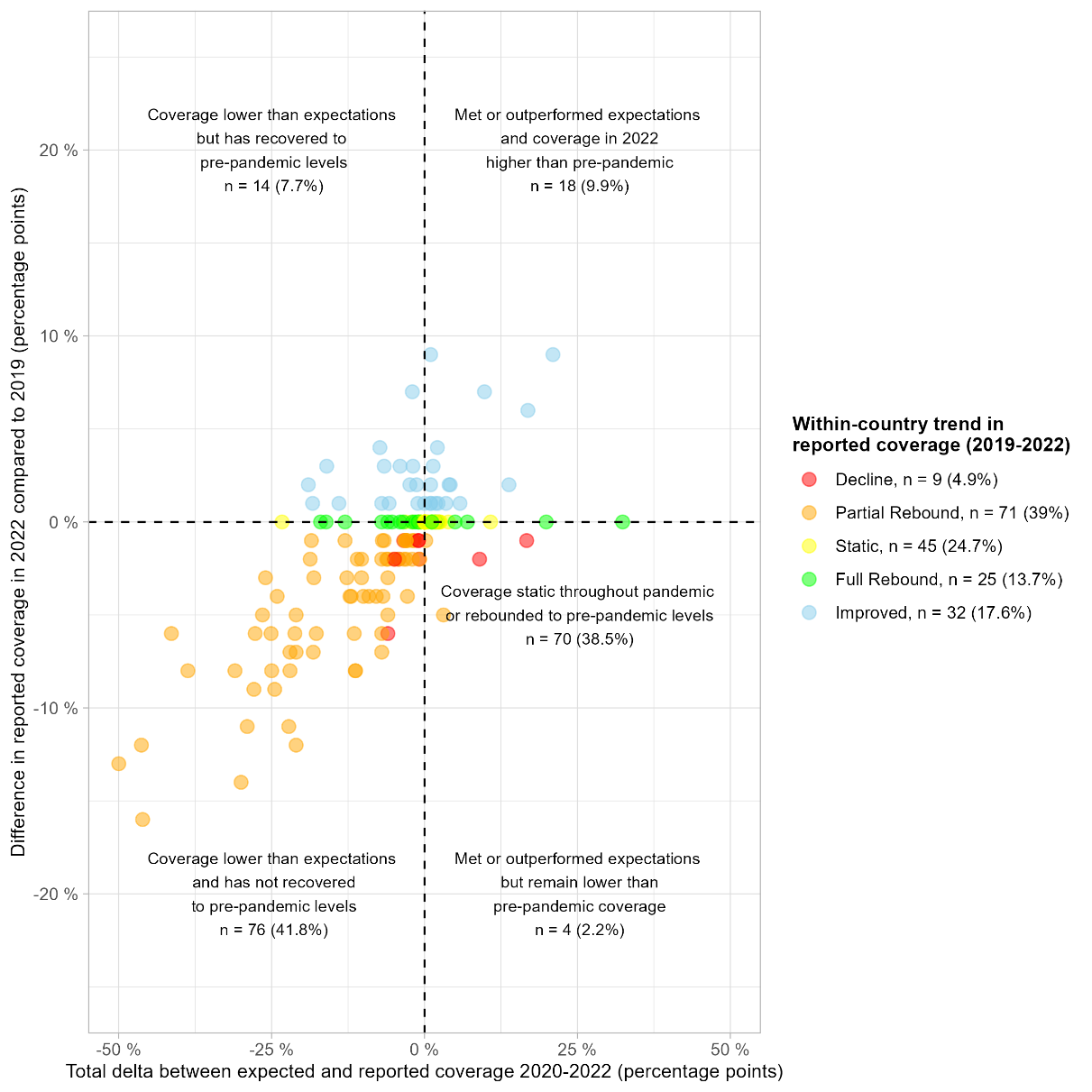


**Figure S10.1 – Classification of pandemic impact and recovery by country for DTP1:** This scatterplot shows the combined 3-year (2020-2022) delta between expected and reported coverage on the x-axis and absolute difference between reported coverage in 2022 and 2019 on the y-axis. Labelling describes and quantifies (total and percentage of 183 countries) country classification with respect to the two plotted dimensions.

1. *MCV1*


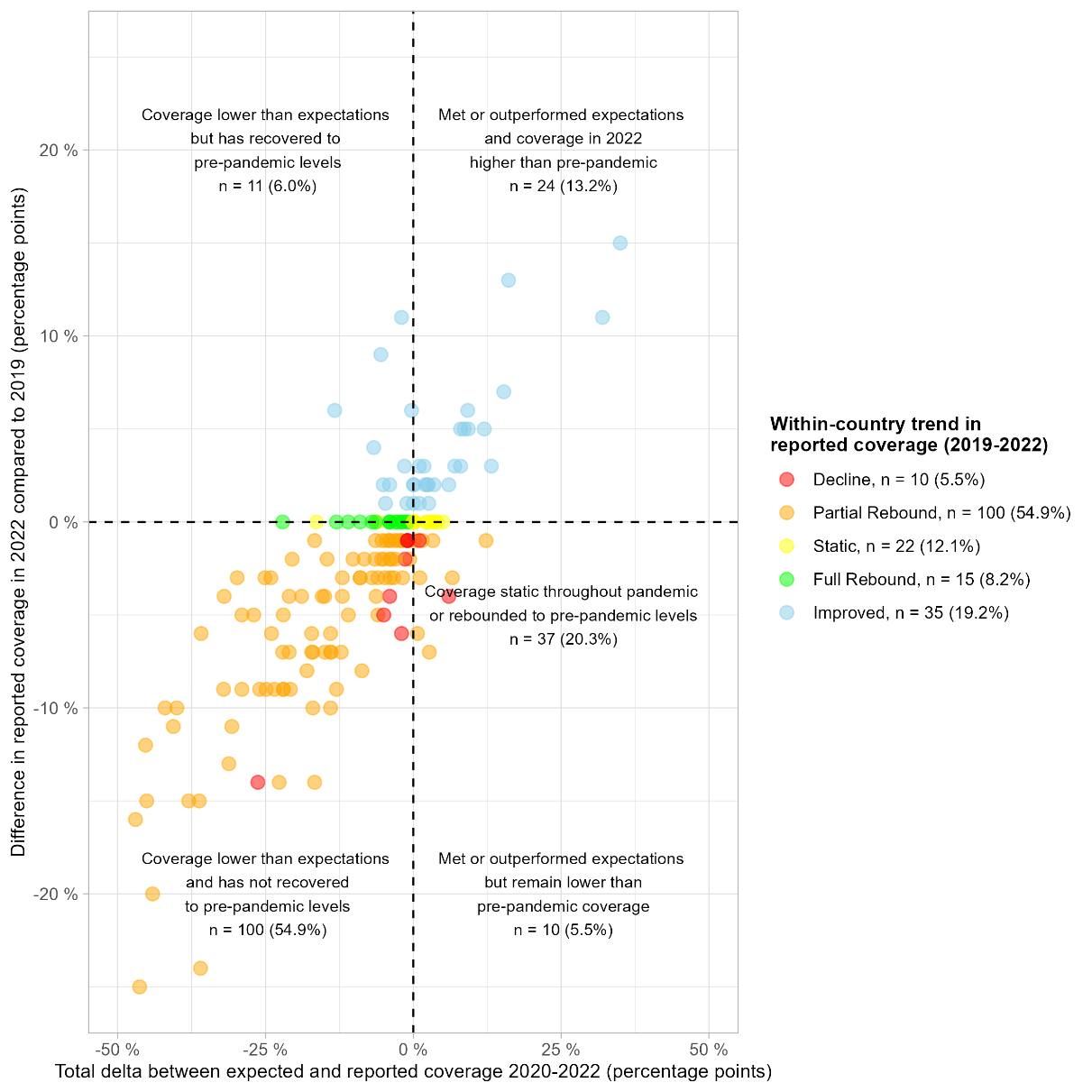


**Figure S10.2 – Classification of pandemic impact and recovery by country for MCV1:** This scatterplot shows the combined 3-year (2020-2022) delta between expected and reported coverage on the x-axis and absolute difference between reported coverage in 2022 and 2019 on the y-axis. Labelling describes and quantifies (total and percentage of 183 countries) country classification with respect to the two plotted dimensions.

**Section S11: Countries classified as improved immunisation performance**

In the manuscript Results, Discussion, and Limitations sections we discuss the challenges with interpreting results for a subset of countries where model fitting appears unreliable. This largely occurs where the ARIMA model selected continues a steep recent coverage trend (e.g., Brazil) or where historic coverage has been highly volatile (e.g., Solomon Islands). Here we show the model fitting for the 10 countries identified as having the greatest performance improvement, as defined by our criteria that quantify absolute and relative performance.

1. DTP1


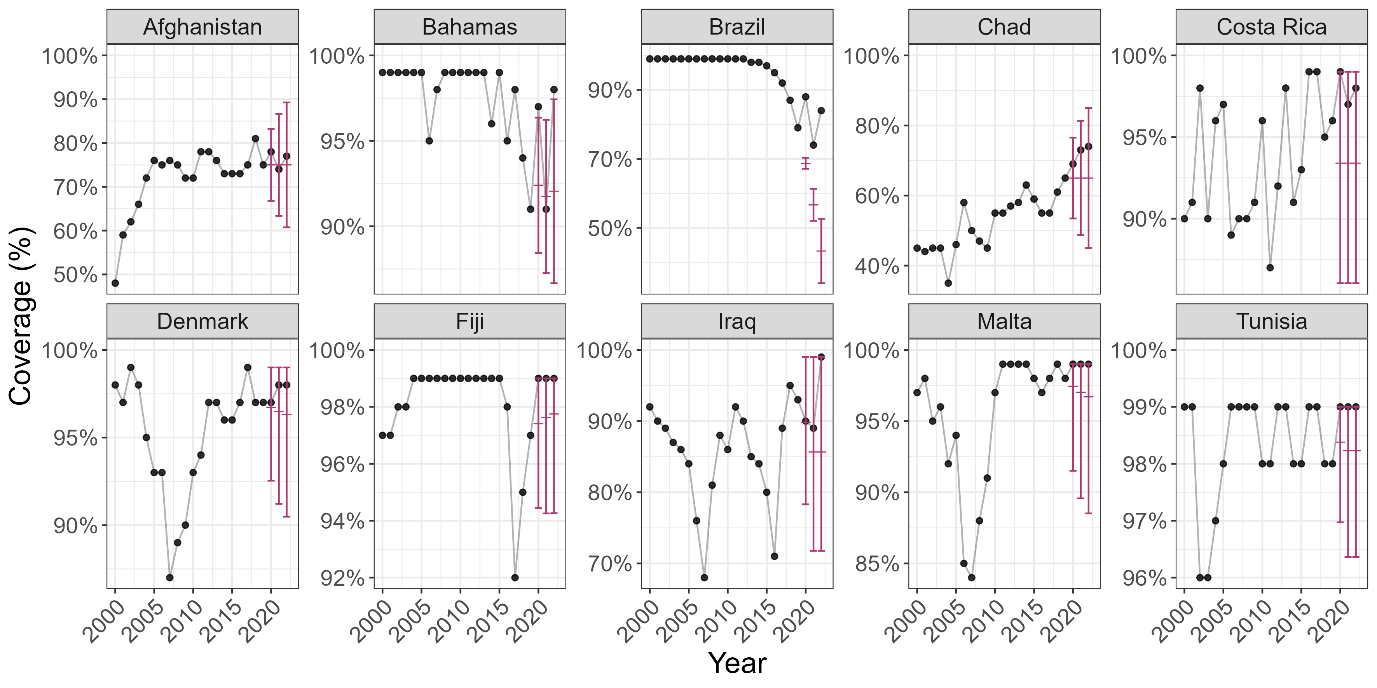


**Figure S11.1 – Visualisation of within-country coverage trends and model fitting for 10 countries with greatest quantified improvement in performance during the pandemic for DTP1:** These graphs show WUENIC-reported coverage data (black dots) from 2000 to 2022 inclusive, and the corresponding ARIMA predictions and the associated 95% CIs (red bars) for 2020 and 2022. Countries included were selected from the top-right corner of analysis shown in Figure S10.1.

1. DTP3


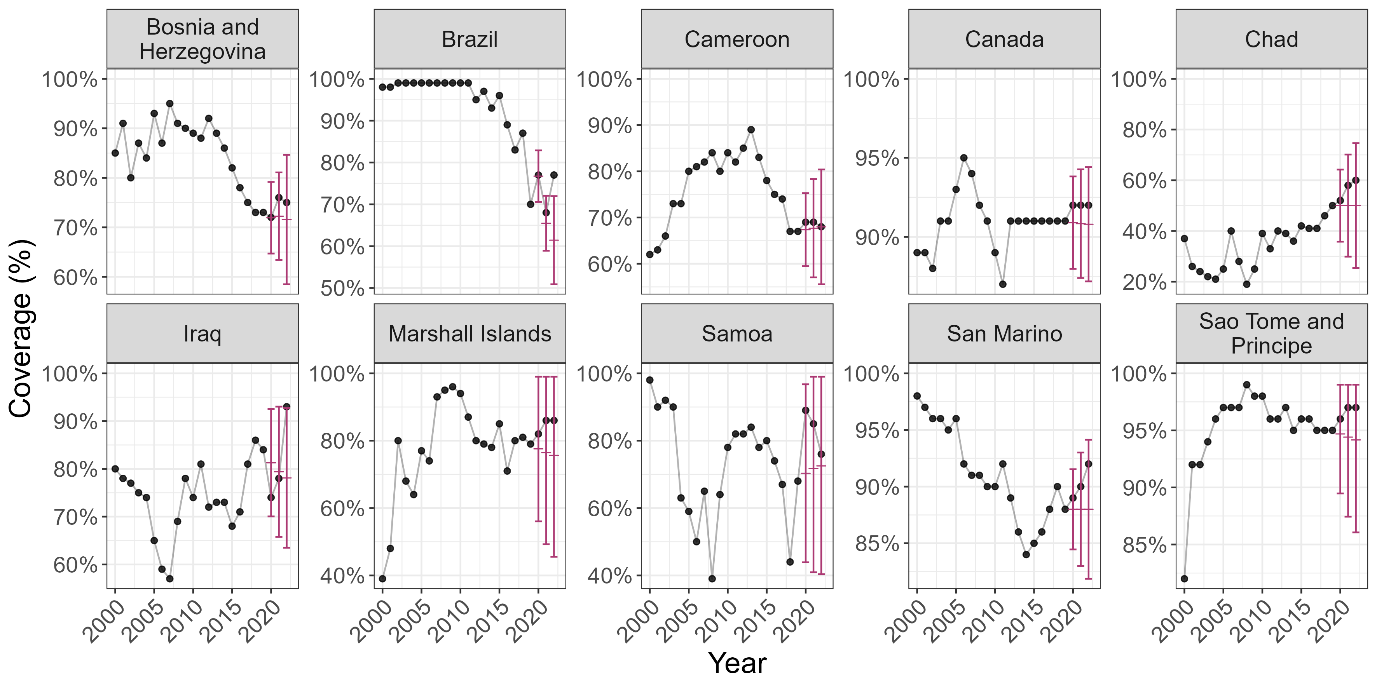


**Figure S11.2 – Visualisation of within-country coverage trends and model fitting for 10 countries with greatest quantified improvement in performance during the pandemic for DTP3:** These graphs show WUENIC-reported coverage data (black dots) from 2000 to 2022 inclusive, and the corresponding ARIMA predictions and the associated 95% CIs (red bars) for 2020 and 2022. Countries included were selected from the top-right corner of analysis shown in manuscript Figure 5.

1. MCV1


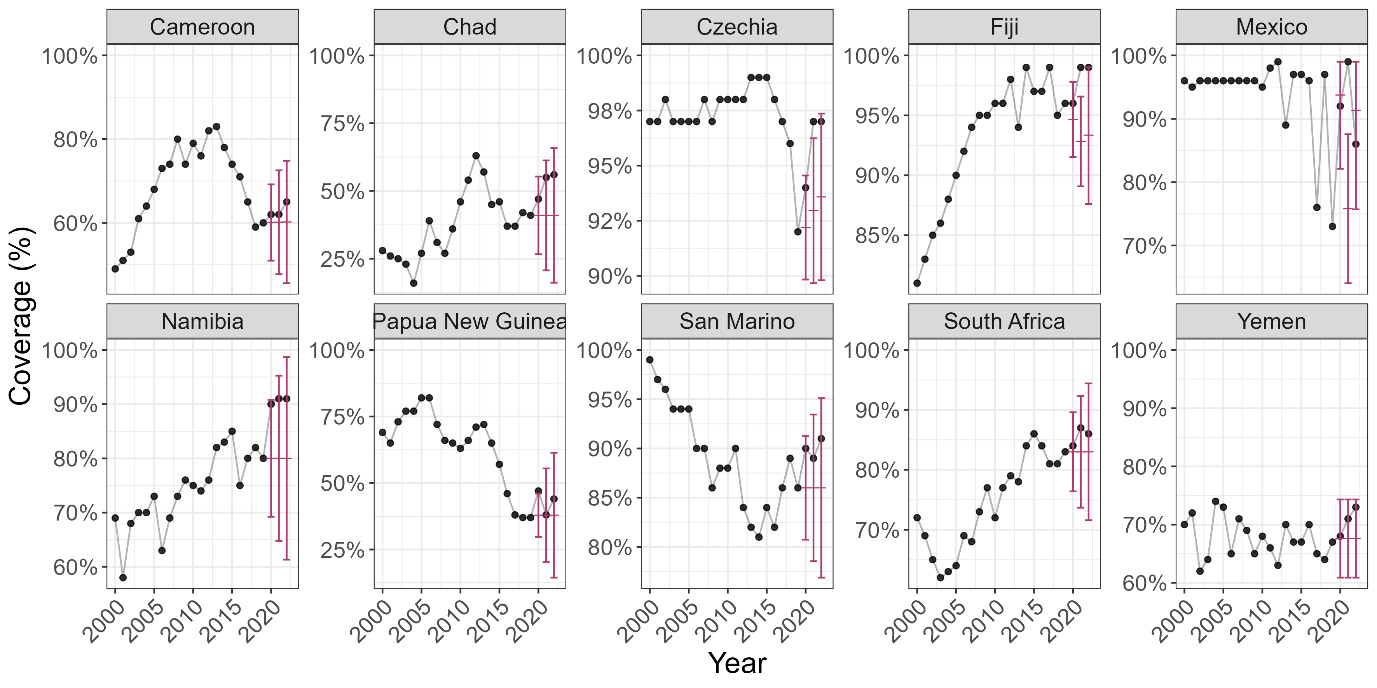


**Figure S11.1 – Visualisation of within-country coverage trends and model fitting for 10 countries with greatest quantified improvement in performance during the pandemic for MCV1:** These graphs show WUENIC-reported coverage data (black dots) from 2000 to 2022 inclusive, and the corresponding ARIMA predictions and the associated 95% CIs (red bars) for 2020 and 2022. Countries included were selected from the top-right corner of analysis shown in Figure S10.2.

**Section S12: Missed immunisations**

1. DTP1 – reported in the manuscript as Figure 6
2. DTP3


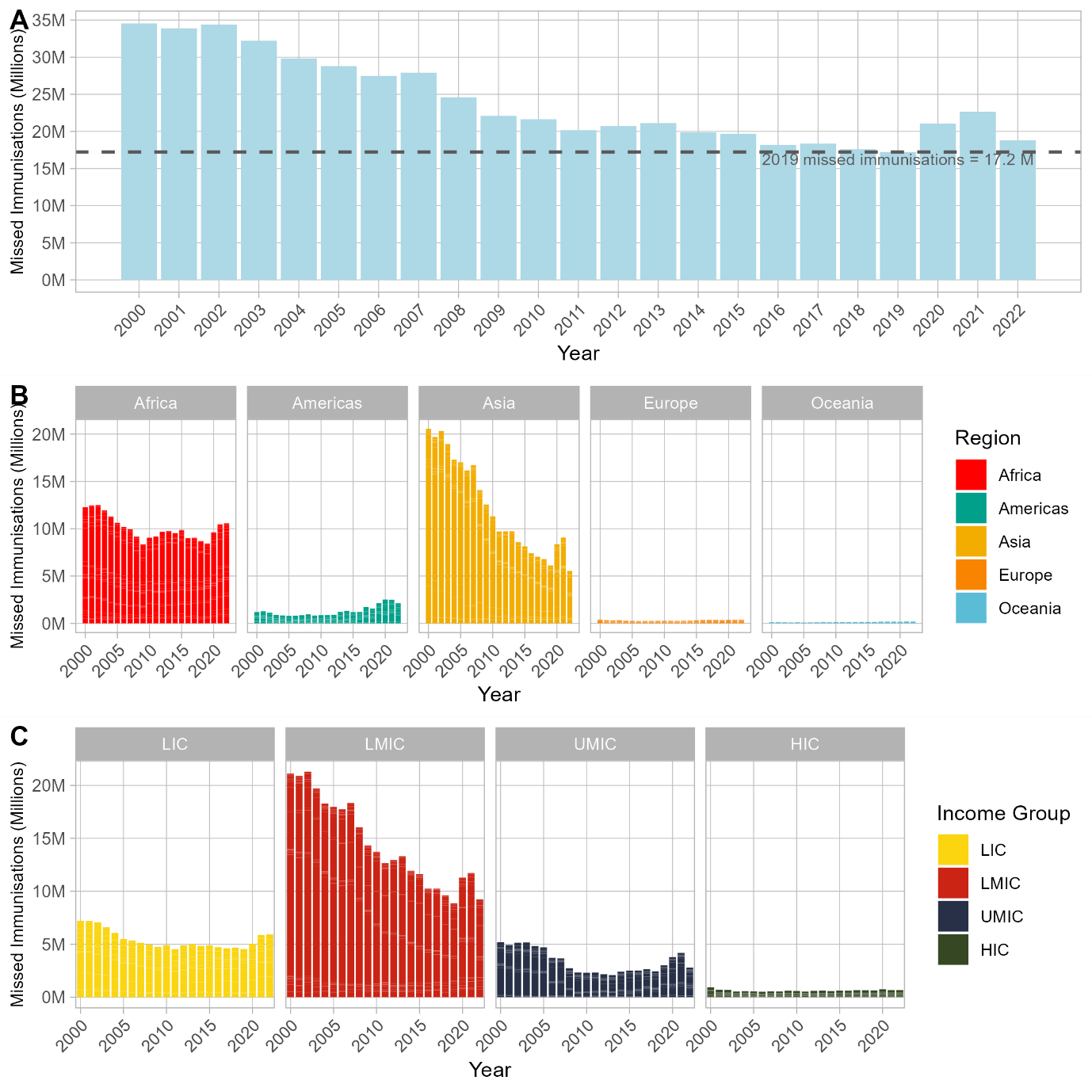


1. MCV1

**
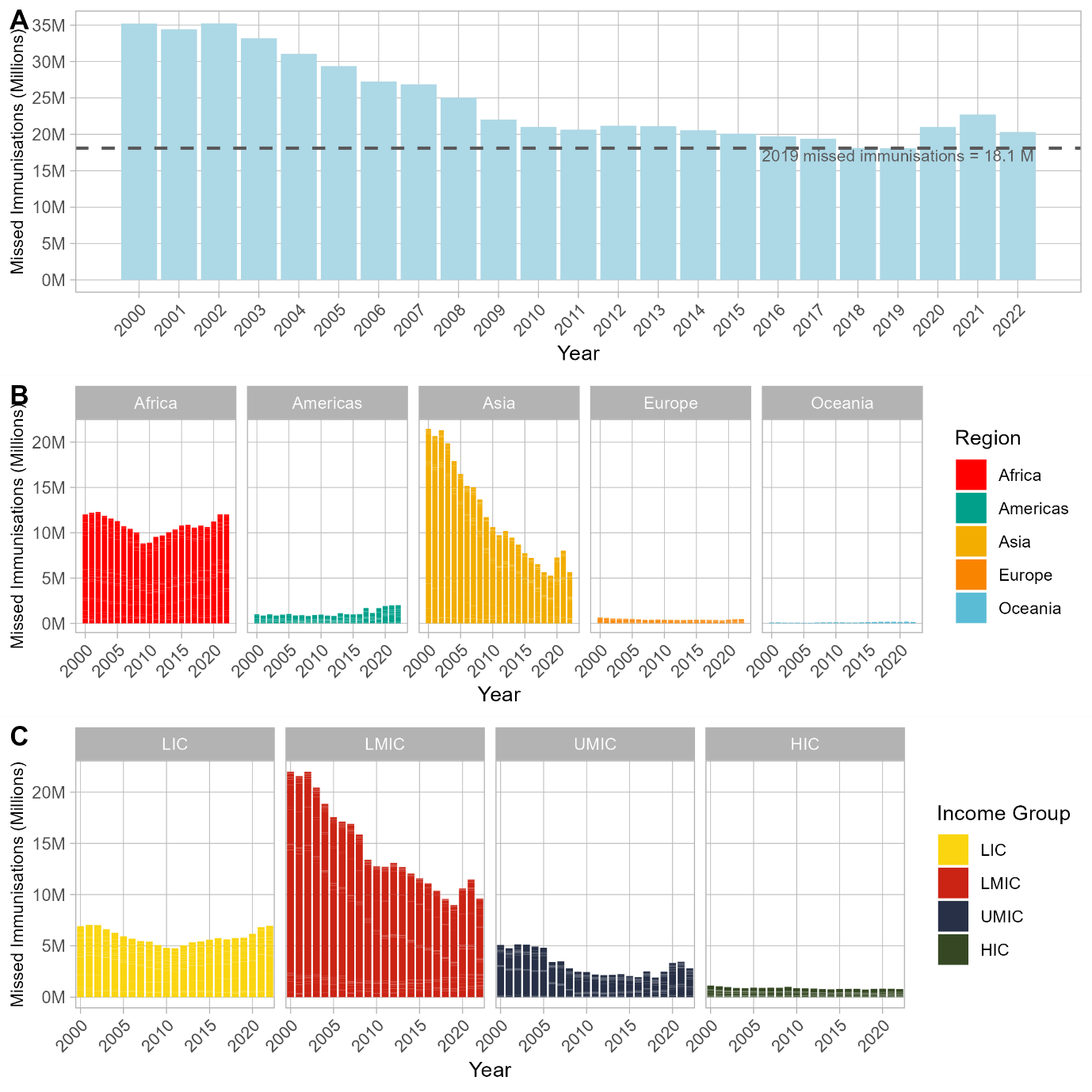
**

**Section S13: Availability and reproducibility**

All analyses were conducted using R 4.1.2 [10]. All the data and R code implementing the analyses are publicly available from the following github repository: <https://github.com/bevans249/modelling_covid_impact_RI>

**References**

[1] K. Harrison, N. Rahimi, and M. Carolina Danovaro-Holliday, “Factors limiting data quality in the expanded programme on immunization in low and middle-income countries: A scoping review,” *Vaccine*, vol. 38, no. 30, pp. 4652–4663, Jun. 2020, doi: 10.1016/J.VACCINE.2020.02.091.

[2] F. T. Cutts, H. S. Izurieta, and D. A. Rhoda, “Measuring Coverage in MNCH: Design, Implementation, and Interpretation Challenges Associated with Tracking Vaccination Coverage Using Household Surveys,” *PLoS Med*, vol. 10, no. 5, p. e1001404, 2013, doi: 10.1371/JOURNAL.PMED.1001404.

[3] N. C. Galles *et al.*, “Measuring routine childhood vaccination coverage in 204 countries and territories, 1980-2019: a systematic analysis for the Global Burden of Disease Study 2020, Release 1,” *Lancet*, vol. 398, no. 10299, pp. 503–521, Aug. 2021, doi: 10.1016/S0140-6736(21)00984-3.

[4] A. Burton, R. Kowalski, M. Gacic-Dobo, R. Karimov, and D. Brown, “A Formal Representation of the WHO and UNICEF Estimates of National Immunization Coverage: A Computational Logic Approach,” *PLoS One*, vol. 7, no. 10, p. e47806, Oct. 2012, doi: 10.1371/JOURNAL.PONE.0047806.

[5] A. Burton *et al.*, “WHO and UNICEF estimates of national infant immunization coverage: methods and processes,” *Bull World Health Organ*, vol. 87, no. 7, pp. 535–541, Jul. 2009, doi: 10.2471/BLT.08.053819.

[6] D. of E. and S. A. P. D. United Nations, “World Population Prospects 2022 Data Sources,” 2022, Accessed: Nov. 20, 2022. [Online]. Available: https://unstats.un.org/unsd/methodology/m49/.

[7] “World Bank Country and Lending Groups – World Bank Data Help Desk.” https://datahelpdesk.worldbank.org/knowledgebase/articles/906519-world-bank-country-and-lending-groups (accessed Nov. 23, 2022).

[8] “countrycode function - RDocumentation.” https://www.rdocumentation.org/packages/countrycode/versions/1.4.0/topics/countrycode (accessed Nov. 29, 2022).

[9] R. J. Hyndman and Y. Khandakar, “Automatic Time Series Forecasting: The forecast Package for R,” *J Stat Softw*, vol. 27, no. 3, pp. 1–22, Jul. 2008, doi: 10.18637/JSS.V027.I03.

[10] R Core Team, “R: A Language and Environment for Statistical Computing,” Vienna, Austria, 2021.
